# Publisher Correction: Atomistic insights into highly active reconstructed edges of monolayer 2H-WSe_2_ photocatalyst

**DOI:** 10.1038/s41467-022-29390-6

**Published:** 2022-03-28

**Authors:** Mohammad Qorbani, Amr Sabbah, Ying-Ren Lai, Septia Kholimatussadiah, Shaham Quadir, Chih-Yang Huang, Indrajit Shown, Yi-Fan Huang, Michitoshi Hayashi, Kuei-Hsien Chen, Li-Chyong Chen

**Affiliations:** 1 Center for Condensed Matter Sciences, National Taiwan University, Taipei, 10617 Taiwan; 2 Center of Atomic Initiative for New Materials, National Taiwan University, Taipei, 10617 Taiwan; 3 Institute of Atomic and Molecular Sciences, Academia Sinica, Taipei, 10617 Taiwan; 4 On leave from Tabbin Institute for Metallurgical Studies, Tabbin, Helwan 109, Cairo, 11421 Egypt; 5 Department of Physics, National Taiwan University, Taipei, 10617 Taiwan; 6 Nano Science and Technology, Taiwan International Graduate Program, Academia Sinica, Taipei, 11529 Taiwan; 7 Institute of Physics, Academia Sinica, Taipei, 11529 Taiwan; 8 Molecular Science and Technology Program, Taiwan International Graduate Program, Academia Sinica, Taipei, 11529 Taiwan; 9 Department of Physics, National Central University, Taoyuan City, 32001 Taiwan; 10 International Graduate Program of Molecular Science and Technology, National Taiwan University, Taipei, 10617 Taiwan; 11 Department of Chemistry, Hindustan Institute of Technology and Science, Chennai, 603103 India

**Keywords:** Photocatalysis, Two-dimensional materials, Photocatalysis, Photocatalysis, Two-dimensional materials, Photocatalysis

Correction to: *Nature Communications* 10.1038/s41467-022-28926-0; Article published online 10 March 2022

The HTML version of this Article included a redundant file ‘Reporting Summary’. This file and related description is now removed.

