# Supplementary material for: Atomistic insights into highly active reconstructed edges of monolayer 2H-WSe2 photocatalyst
Source: Nat Commun. 2022 Mar 10;13:1256. doi: 10.1038/s41467-022-28926-0 (PMC8913837; doi:10.1038/s41467-022-28926-0)
Supplement: Supplementary file 1 — Supplementary Information [file 41467_2022_28926_MOESM1_ESM.pdf]

SUPPLEMENTARY INFORMATION

Atomistic insights into highly active reconstructed edges of monolayer 2H-WSe_2 photocatalyst

Mohammad Qorbani[1,2], Amr Sabbah[3,4], Ying-Ren Lai[1,2], Septia Kholimatussadiah[1,5,6,7], Shaham Quadir[1,3,8,9], Chih-Yang Huang[1,3,8,10], Indrajit Shown[3,11], Yi-Fan Huang[3], Michitoshi Hayashi[1,2], Kuei-Hsien Chen[1,3]✉ & Li-Chyong Chen[1,2,5]✉

[1]Center for Condensed Matter Sciences, National Taiwan University, Taipei 10617, Taiwan.

[2]Center of Atomic Initiative for New Materials, National Taiwan University, Taipei 10617, Taiwan.

[3]Institute of Atomic and Molecular Sciences, Academia Sinica, Taipei 10617, Taiwan.

[4]On leave from Tabbin Institute for Metallurgical Studies, Tabbin, Helwan 109, Cairo 11421, Egypt.

[5]Department of Physics, National Taiwan University, Taipei 10617, Taiwan.

[6]Nano Science and Technology, Taiwan International Graduate Program, Academia Sinica, Taipei 11529, Taiwan.

[7]Institute of Physics, Academia Sinica, Taipei, 11529, Taiwan.

[8]Molecular Science and Technology Program, Taiwan International Graduate Program, Academia Sinica, Taipei 11529, Taiwan.

[9]Department of Physics, National Central University, Taoyuan City 32001, Taiwan.

[10]International Graduate Program of Molecular Science and Technology, National Taiwan University, Taipei 10617, Taiwan.

[11]Department of Chemistry, Hindustan Institute of Technology and Science, Chennai 603103, India.

These authors contributed equally: Amr Sabbah and Ying-Ren Lai.

These authors jointly supervised this work: Kuei-Hsien Chen and Li-Chyong Chen.

✉chenkh@pub.iams.sinica.edu.tw; chenlc@ntu.edu.tw

Correspondence and requests for materials should be addressed to K.-H. C. or to L.-C. C.

Contents

Supplementary Figures 5

Supplementary Fig. 1 Strategies to enhance the efficiency of the gas-phase PC CO_2RR. 5

Supplementary Fig. 2 Height profile of the monolayer WSe_2. 6

Supplementary Fig. 3 Lattice vibrational modes of ML WSe_2. 6

Supplementary Fig. 4 Raman spectra of the ML WSe_2 with different sizes. 7

Supplementary Fig. 5 Raman spectra of the ML WSe_2 at different excitation powers. 8

Supplementary Fig. 6 Photoluminescence spectra of the ML WSe_2 at room temperature. 9

Supplementary Fig. 7 Laser power-dependent PL intensity of the ML WSe_2. 10

Supplementary Fig. 8 Exciton and trion dynamics of ML WSe_2. 11

Supplementary Fig. 9 Time-resolved PL experiments. 12

Supplementary Fig. 10 Comparison between ML WSe_2 grown by vapor deposition and prepared by micromechanical exfoliation. 13

Supplementary Fig. 11 Temperature-dependent PL peak positions. 14

Supplementary Fig. 12 Microstructure and selected-area electron diffraction pattern of the ML WSe_2. 15

Supplementary Fig. 13 Microstructure of basal plane of ML WSe_2 flake. 16

Supplementary Fig. 14 Intrinsic point defects by density functional theory calculations. 17

Supplementary Fig. 15 Thermodynamic stability of the various intrinsic defects in the basal plane. 18

Supplementary Fig. 16 Electronic properties of the basal plane with the intrinsic defects. 19

Supplementary Fig. 17 Edge reconstruction by DFT calculation. 19

Supplementary Fig. 18 Edge configurations with defects by DFT calculation. 20

Supplementary Fig. 19 Electronic properties of the reconstructed edges. 21

Supplementary Fig. 20 CO_2 adsorption on the basal plane of ML WSe_2. 22

Supplementary Fig. 21 CO_2 adsorption at the regular reconstructed edges of ML WSe_2. 23

Supplementary Fig. 22 CO_2 adsorption at the defective reconstructed edges of ML WSe_2. 24

Supplementary Fig. 23 Schematic illustration of AFM-SECM mechanism. 25

Supplementary Fig. 24 SECM feedback mode. 26

Supplementary Fig. 25 Photocatalyst yields and blank tests. 27

Supplementary Fig. 26 Carbon monoxide yield. 28

Supplementary Fig. 27 Oxidation product. 29

Supplementary Fig. 28 Simulated yield of PC CO_2RR. 30

Supplementary Tables 31

Supplementary Table 1 Examples of different materials for PC production of CO_2. 31

Supplementary Table 2 Turnover frequency and consumed electron rate. 33
Supplementary Notes 34
Supplementary Note 1. Growth recipe 34
1–1. Low-pressure vapor deposition. 34
Supplementary Note Fig. 1 Illustration of the growth recipe of low-pressure vapor deposition. 35
Supplementary Note Fig. 2 Photoluminescence spectra of the ML and BL WSe_2. 36
Supplementary Note Fig. 3 Uniformity of the number of layers by photoluminescence. 37
Supplementary Note Fig. 4 Growth of the partial second layer. 38
1–2. Atmospheric-pressure chemical vapor deposition. 38
Supplementary Note 2. Monolayer WSe_2 transfer 39
Supplementary Note 3. Characterizations 39
3–1. Analyzing optical microscopy images. 39
3–2. Fitting X-ray photoelectron spectroscopy spectra. 39
3–3. Photoluminescence and Raman experiments. 40
3–4. Overall absorption percentage of ML WSe_2. 40
Supplementary Note 4. Density functional theory calculations 41
4–1. Computational method. 41
4–2. Models of basal plane. 42
4–3. Models of regular edges. 42
Supplementary Note Fig. 5 Regular edge by DFT calculation. 43
4–4. Models of defective edges. 43
Supplementary Note Table 1 Models of defective edges. 44
4–5. CO_2 adsorption calculation. 44
4–6. Calculation of formation energy. 45
4–7. Convergence of adsorption and formation energy. 46
Supplementary Note Fig. 6 CO_2 adsorption on the basal plane and edges of ML WSe_2. 48
Supplementary Note Table 2 The convergence test for the defect formation energies. 49
Supplementary Note Table 3 A comparison of the in-plane defect formation energies between the nanoribbon and monolayer models. 49
Supplementary Note 5. Ag photodeposition and nanoscale redox mapping. 49
5–1. Photodeposition of Ag nanoparticles. 49
5–2. Atomic force microscopy-scanning electrochemical microscopy. 49
Supplementary Note Fig. 7 AFM-SECM feedback mapping of Pt strands separated by square Si_3N_4 islands. 51

Supplementary Note 6. Photocatalytic CO_2 reduction 52
6–1. Photocatalytic setup. 52
Supplementary Note Fig. 8 Gas chromatography–mass spectrometry of the CH_4 product. 52
6–2. Calculation of internal quantum efficiency. 53
6–3. Calculation of consumed electron rates. 53
Supplementary Note 7. Illustration of the relaxed configurations 54
Supplementary References 54
Reference 1 to 58

Supplementary Figures

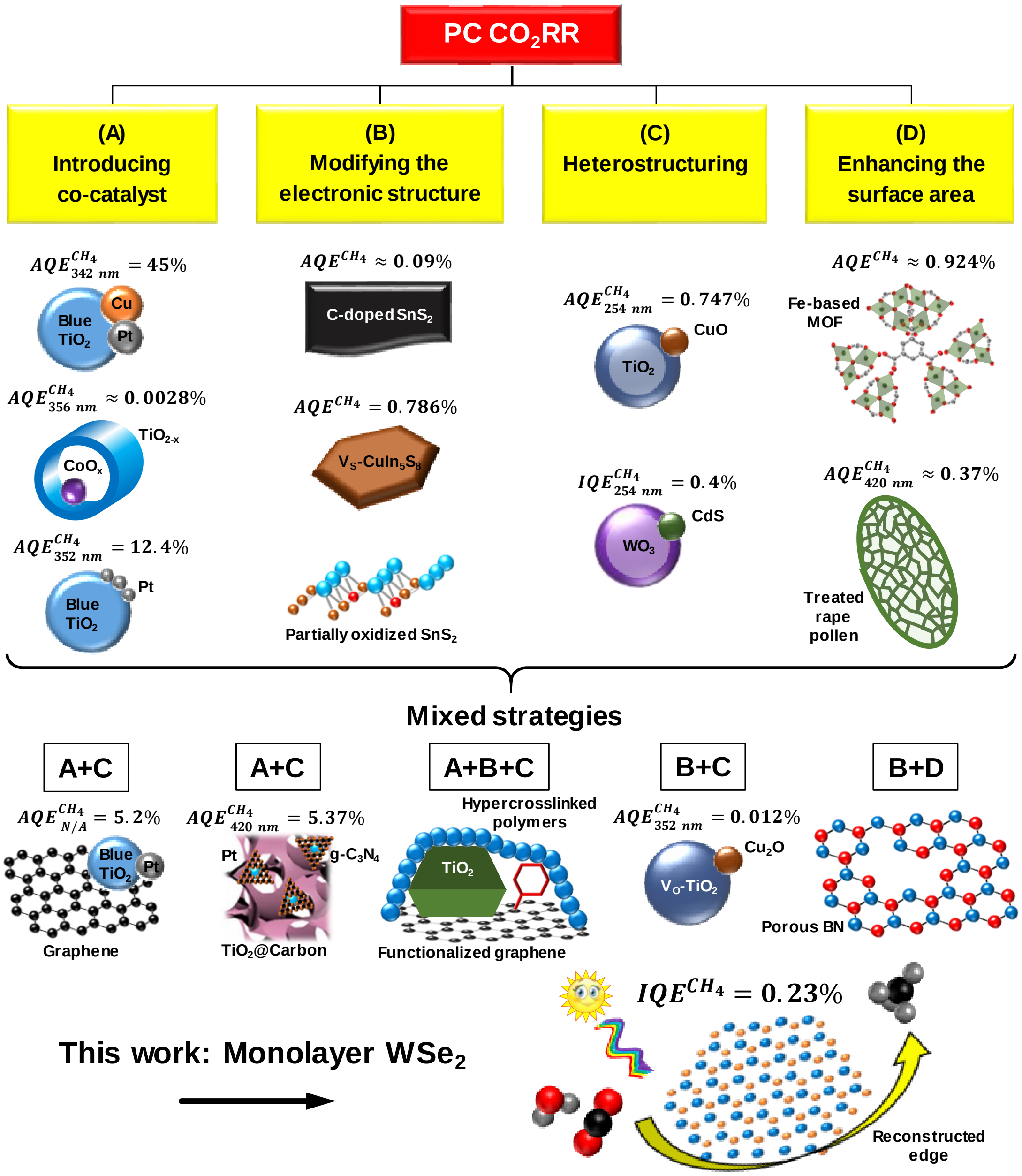

Supplementary Fig. 1 Strategies to enhance the efficiency of the gas-phase PC CO_2RR. There are several strategies to increase the CO_2 conversion yield include (A) Introducing co-catalyst, (B) Modifying the electronic structure, (C) Heterostructuring, and (D) Enhancing the surface area. Besides, mixed strategies can also help to boost the conversion rate. More details about the materials and references are summarized in Supplementary Table 1.

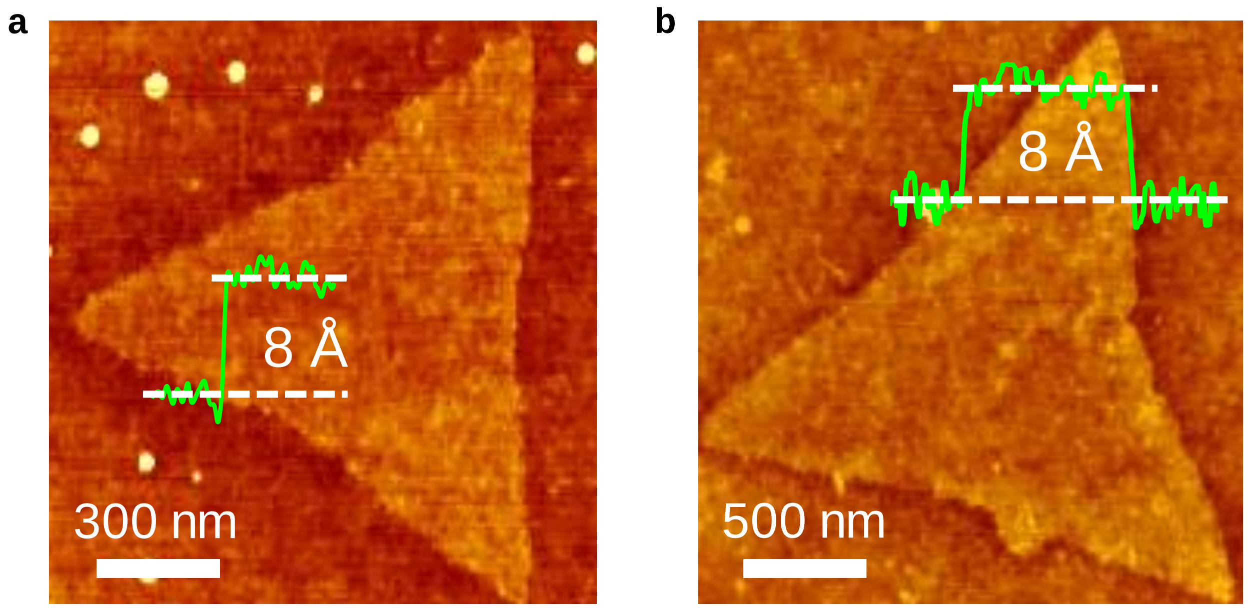

Supplementary Fig. 2 Height profile of the monolayer WSe_2. Atomic force microscope (AFM) images for two different monolayer (ML) WSe_2 flakes with perimeters of ~2.2 μm (left panel) and ~4.5 μm (right panel).

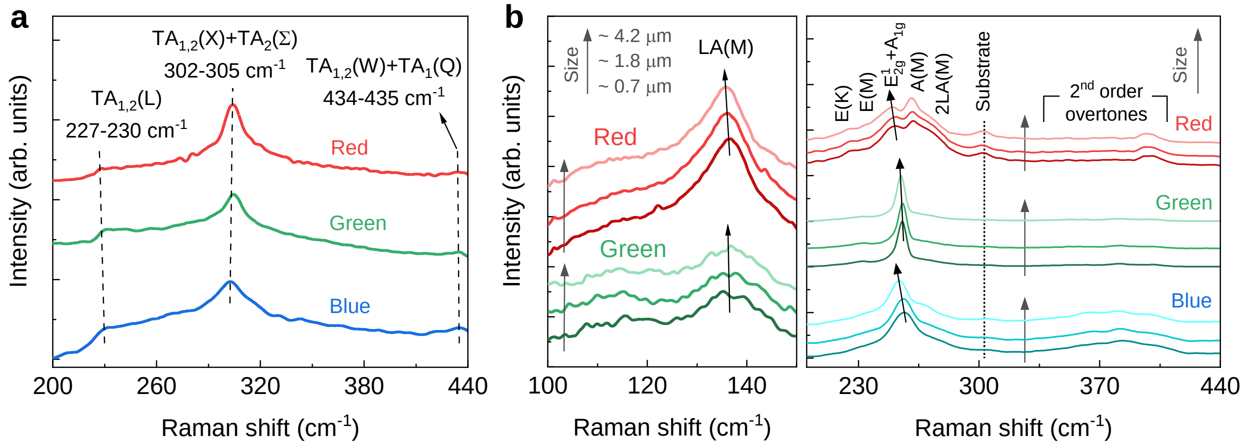

Supplementary Fig. 3 Lattice vibrational modes of ML WSe_2. Raman spectra of the **a** SiO_2/Si substrate and **b** ML WSe_2 using different laser excitation wavelengths of 473, 532, and 632 nm, named blue, green, and red, respectively. For each laser excitation wavelength, Raman spectra taken from samples with different sizes (specifically, 0.7 μm, 1.8 μm, and 4.2 μm) are displayed in gray color. The vibrational modes of the SiO_2/Si substrate are added based on the previous reports[1,2].

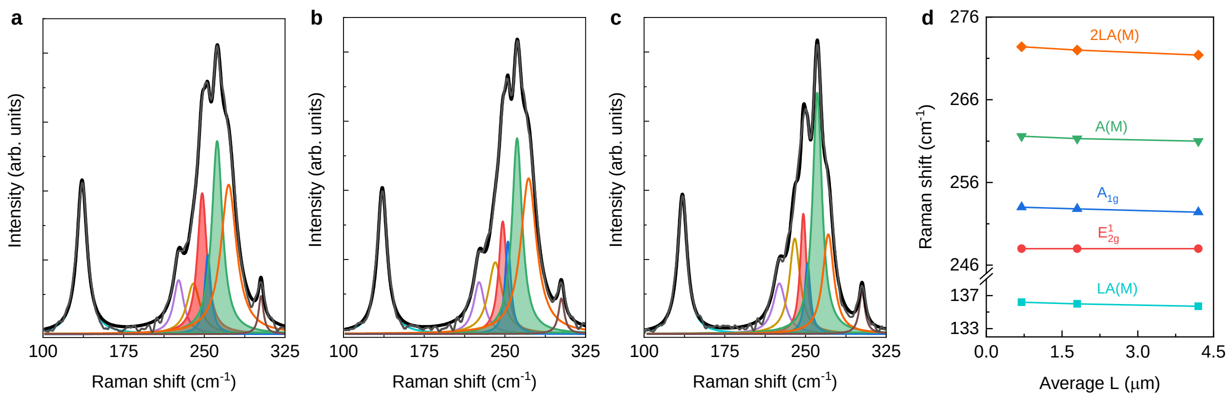

Supplementary Fig. 4 Raman spectra of the ML WSe_2 with different sizes. Raman spectra from **a** $L \approx 0.7$ μm; **b** $L \approx 1.8$ μm; and **c** $L \approx 4.2$ μm using a laser excitation wavelength of 632 nm (red laser). Lorentzian fitting functions were used to fit the spectra resulted in the following vibrational modes (in order of increasing Raman shift positions): $LA(M)$, $E(K)$, $E(M)$, E_{2g}^{1}, A_{1g}, $A(M)$, $2LA(M)$ for ML WSe_2, and $TA_{1.2}(X) + TA_2(\Sigma)$ at 303.5 cm^{-1} for SiO_2/Si substrate from left to right. **d** Raman-active modes vs. the average lateral size (characteristic size) of the flakes. It is also observed that the peak of the degenerated $E_{2g}^{1} + A_{1g}$ modes slightly blue-shifted and broadened with decreasing L. The $E(K)$, $E(M)$, and $TA_{1.2}(X) + TA_2(\Sigma)$ peaks colored in violent, dark yellow, and dark purple, respectively, do not draw for the sake of presentation.

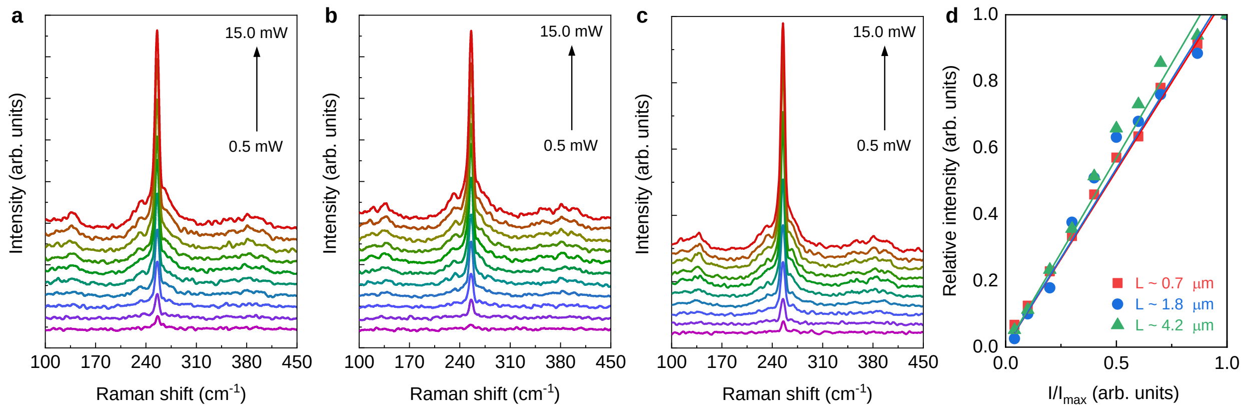

Supplementary Fig. 5 Raman spectra of the ML WSe_2 at different excitation powers. Raman spectra from **a** $L \approx 0.7$ µm; **b** $L \approx 1.8$ µm; and **c** $L \approx 4.2$ µm for different laser powers using excitation wavelengths of 532 nm (green laser). **d** relative $E_{2g}^{1} + A_{1g} + A(M)$ peak intensity vs. relative laser power, I/I_{max}. Usually, laser radiation shifts the vibrational modes of the ML TMDCs[3]. In the present study, the invariableness of the peak positions and linear power-dependent peak intensities for the different excitation powers show that the utilized range of laser power is insufficient for heating the samples.

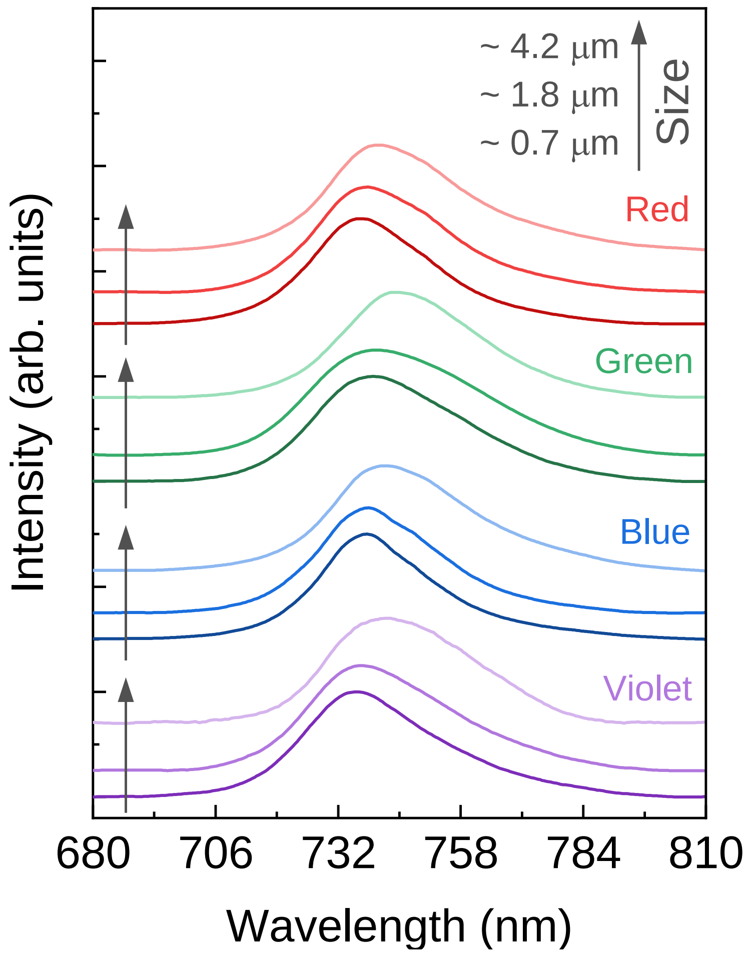

Supplementary Fig. 6 Photoluminescence spectra of the ML WSe_2 at room temperature. Photoluminescence (PL) spectra of the flakes with different sizes ($L \approx 0.7$ μm, $L \approx 1.8$ μm, and $L \approx 4.2$ μm) and laser excitation wavelengths. The non-symmetric curves are fitted with two Gaussian peaks corresponding to the neutral exciton (X^0) and charged exciton (or trion; X^-) quasiparticles[4].

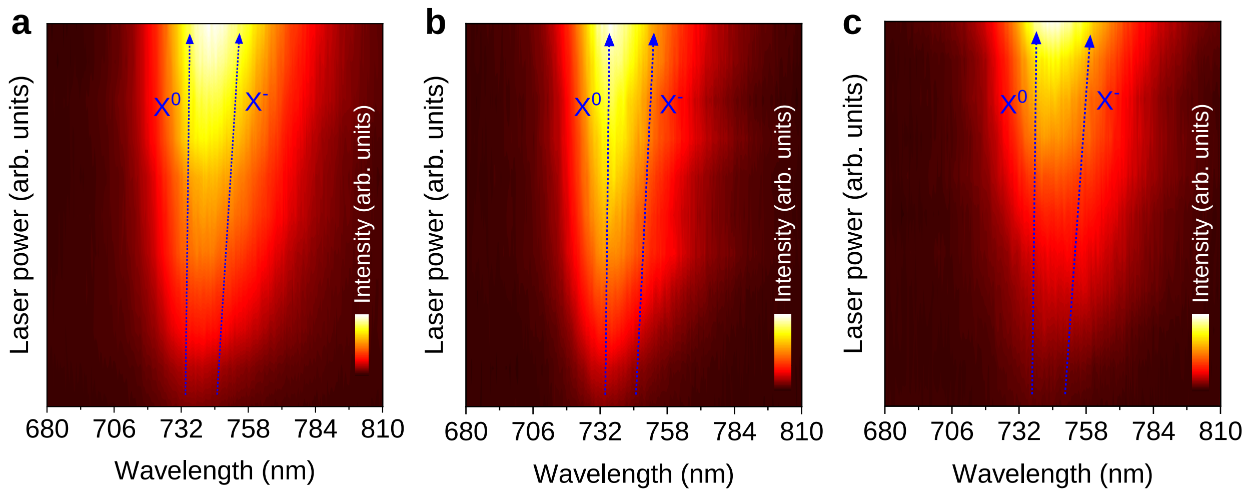

Supplementary Fig. 7 Laser power-dependent PL intensity of the ML WSe_2. PL intensity for different sizes of **a** $L \approx 0.7$ µm, **b** $L \approx 1.8$ µm, and **c** $L \approx 4.2$ µm, respectively, using a laser excitation wavelength of 405 nm (blue laser). The PL peaks, for both X^0 and X^-, red-shift with increasing laser power. The room-temperature PL spectra reveal that the optical band gap (X^0 peak position) of the ML flakes is in the range of 733 to 738 nm (i.e. 1.68 eV), almost independent of the laser power.

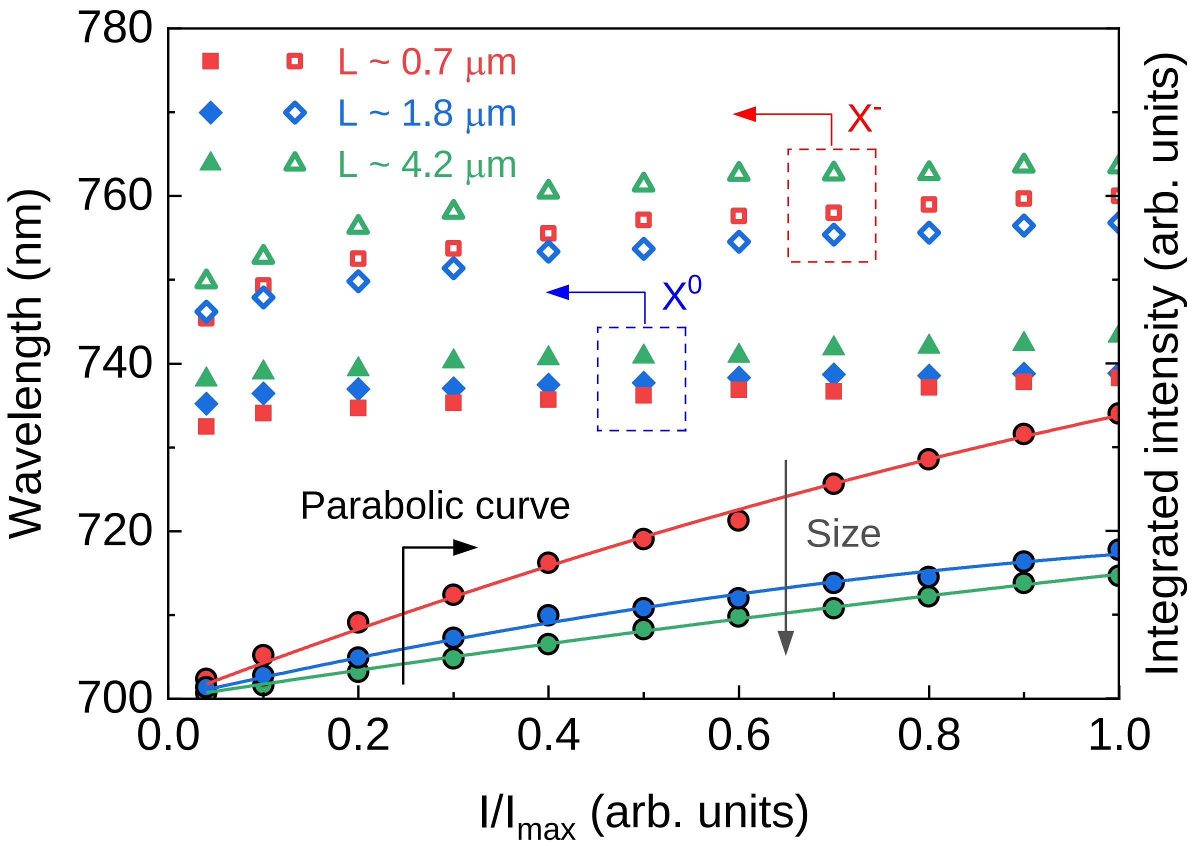

Supplementary Fig. 8 Exciton and trion dynamics of ML WSe₂. X^0, X^- peak positions, and integrated intensity of the PL peak vs. I/I_{max} at room temperature. The PL peaks, for both X^0 and X^-, red-shift with increasing laser power. We found that the integrated intensity of the PL emission depicts a parabolic increase as a function of the laser power. The concurrent red-shift and non-linear enhancement of the PL emission can be attributed to band structure renormalization due to plasma screening and phase space filling[5]. With the generation of a sufficient number of charge carriers, the position of the Λ point lowers faster than the K point of the Brillouin zone. It results in electron accumulation at Λ point and red-shift of the X^- peak ($\Delta E_{X^0\text{-}X^-} \approx 45$ meV) at high excitation power.

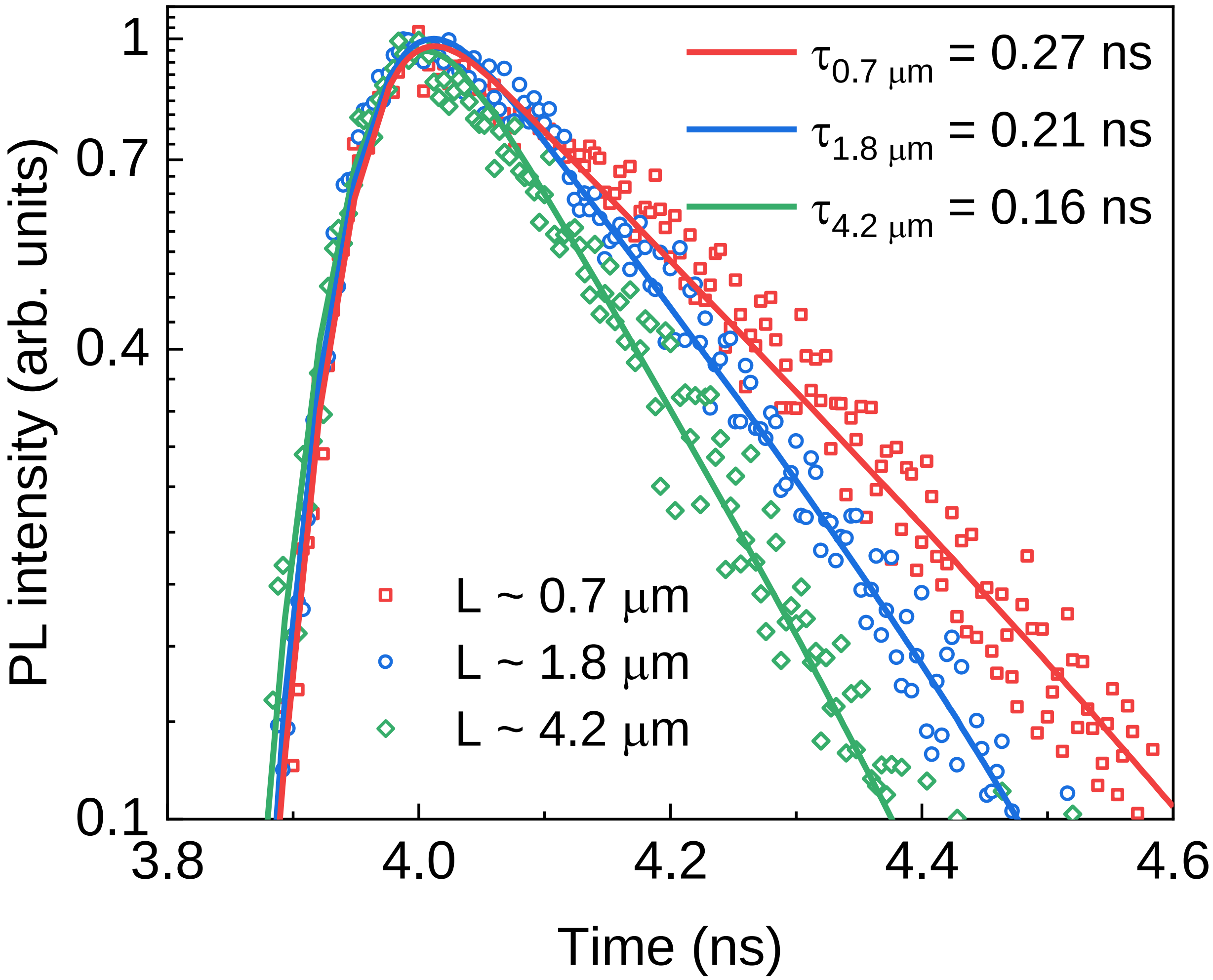

Supplementary Fig. 9 Time-resolved PL experiments. Normalized PL intensities for different sizes of ML WSe_2 flakes, respectively.

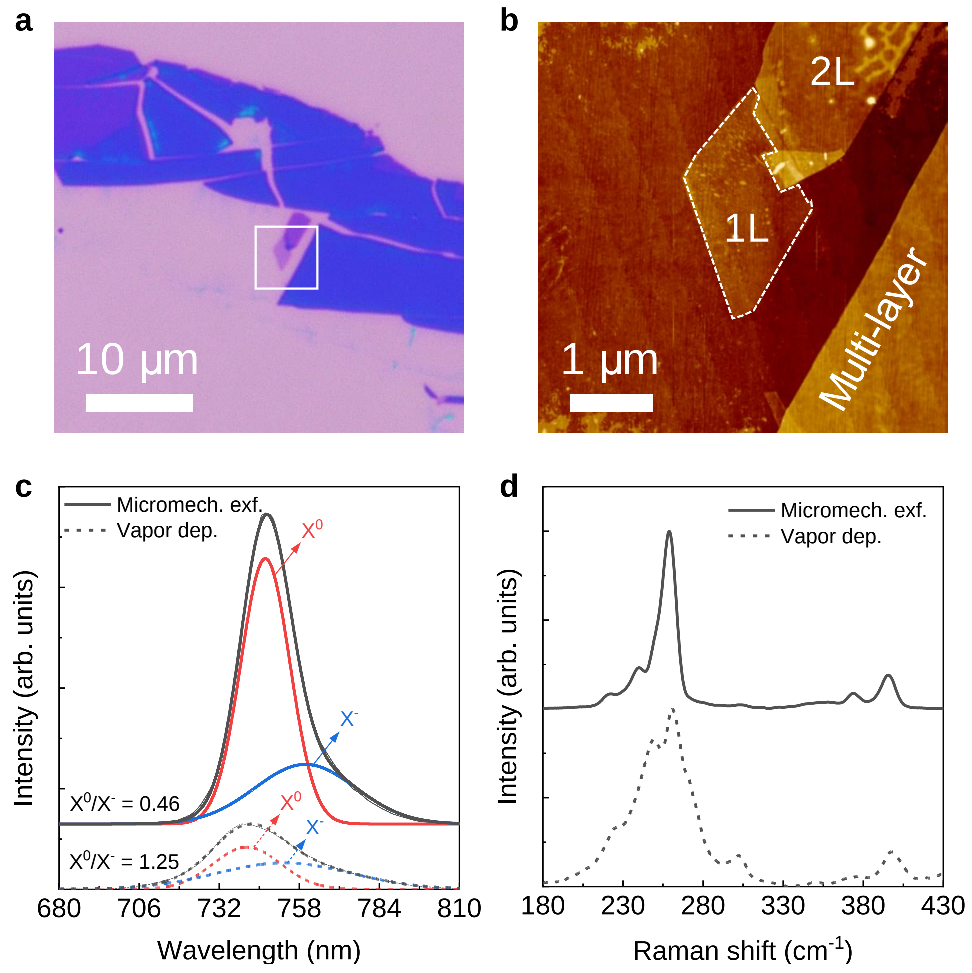

Supplementary Fig. 10 Comparison between ML WSe_2 grown by vapor deposition and prepared by micromechanical exfoliation. a Optical microscope and **b** AFM height profile images, respectively. **c** room-temperature PL and **d** Raman spectra, respectively, were recorded by using a red laser (632 nm). the PL intensity of the VT-WSe_2 (grown by low-pressure vapor deposition) is much lower than that of the ME-WSe_2 (prepared by a micromechanical exfoliation) due to the presence of mid-gap states and a high non-radiative recombination rate. In addition, the high X^{-}–to–X^{0} ratios are 0.46 and 1.25 for the ME-WSe_2 and VT-WSe_2, respectively. This implies that the presence of localized charge carriers is due to the higher defect density in the VT-WSe_2 sample. In contrast to VT-WSe_2, the Raman spectrum of the ME-WSe_2 shows an intense $A(M)$ peak with a small shoulder at ~250 cm^{-1} ($E_{2g}^{1} + A_{1g}$).

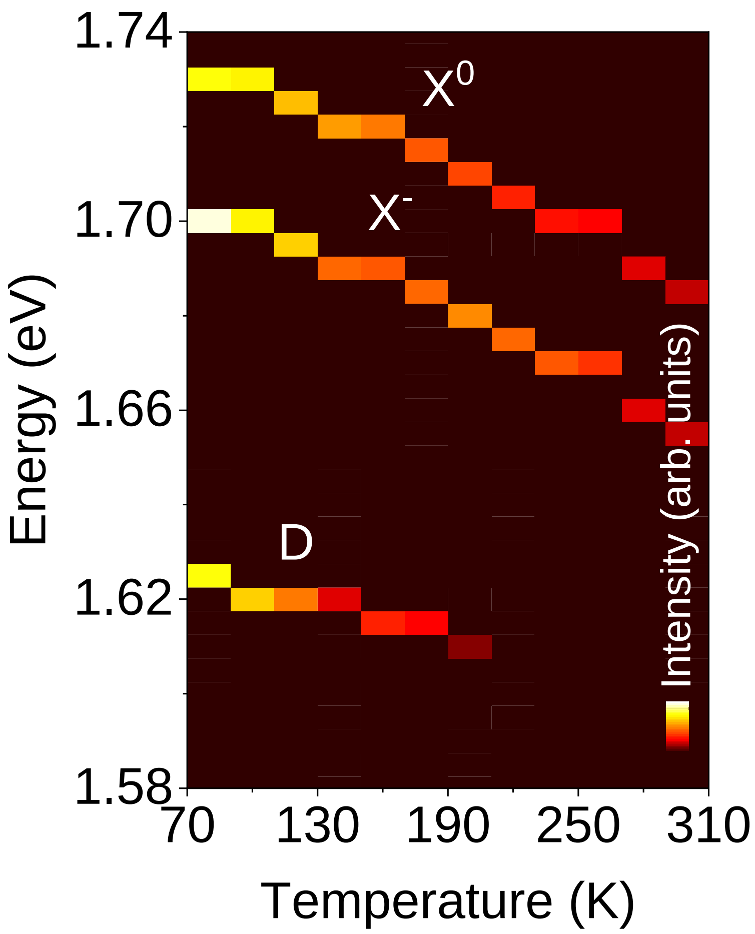

Supplementary Fig. 11 Temperature-dependent PL peak positions. Temperature-dependent PL peak positions of the exciton (X^0), trion (X^-), and defect (D) states, respectively. The peaks are fitted with the Bose–Einstein statistical function (not shown here). Electron-phonon coupling constant and average phonon temperature are calculated of 18 meV and 165 K (14.2 meV), respectively[6,7].

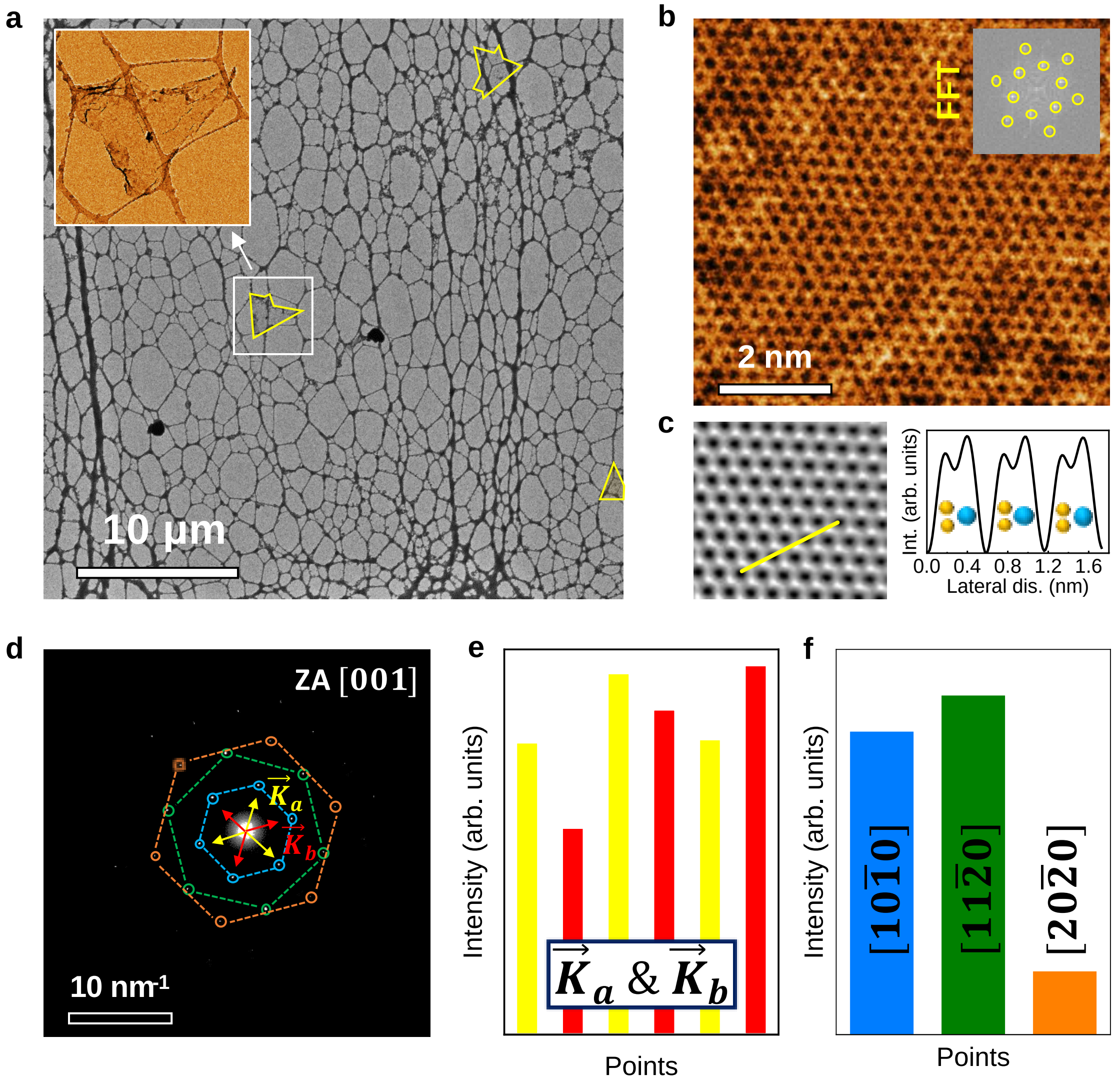

Supplementary Fig. 12 Microstructure and selected-area electron diffraction pattern of the ML WSe_2. a Low-magnification TEM image of the transferred flakes on the lacey supported Cu grid. **b** HRTEM image of the basal plane. The inset shows the corresponding fast Fourier transform (FFT) pattern. It reveals the hexagonal lattice of the ML WSe_2. **c** Filtered HRTEM image. W and Se elements can be distinguished based on their intensity as shown by the yellow line. **d** Typical selected-area electron diffraction (SAED) pattern of the ML crystal at zone axis of [001]. **e** Intensity of the six $[10\bar{1}0]$ diffraction spots: Yellow and red colors are for $\vec{K}_a$ and $\vec{K}_b$ spots, respectively. **f** Average intensity of the diffraction spots for the first three hexagons. $\vec{K}_a$ spots are 5% to 10% brighter than $\vec{K}_b$ spots due to W and Se sublattices with three-fold symmetry, respectively[8].

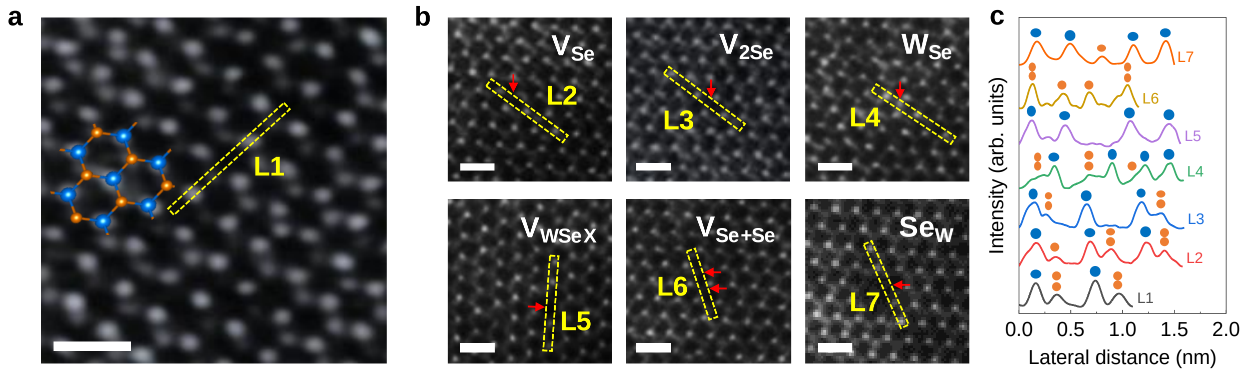

Supplementary Fig. 13 Microstructure of basal plane of ML WSe_2 flake. a HAADF-STEM images of the basal plane and **b** typical observable intrinsic point defects. The brightest and dimmer spots are assigned to W and Se atoms, respectively. Several point defects including V_{Se}, V_{2Se}, W_{Se}, V_{WSeX}, V_{Se+Se}, Se_W exist in the basal plane. Blue- and orange-filled circles are W and Se atoms, respectively. Red arrows show the locations of the defects. Scale bars are 0.5 nm. **c** HAADF intensity profiles along the corresponding lines in **b**.

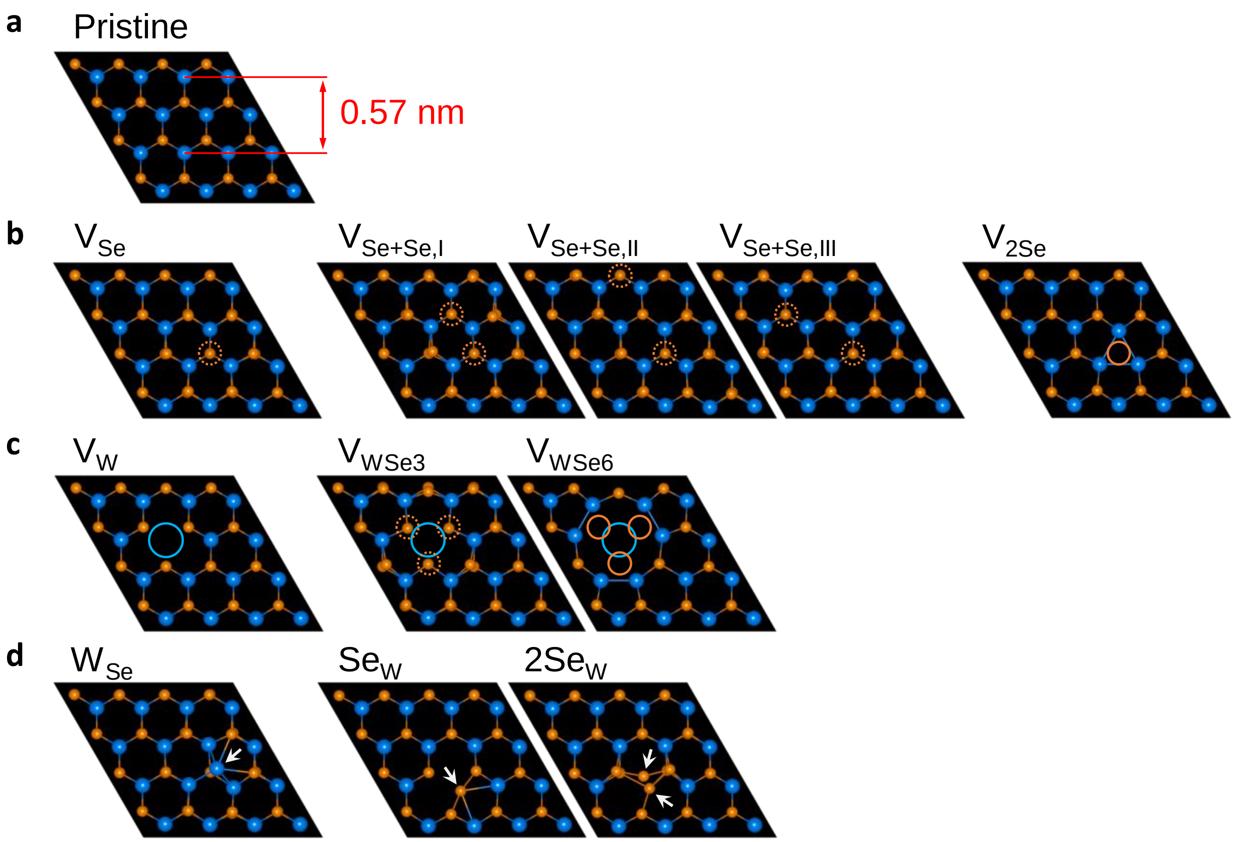

Supplementary Fig. 14 Intrinsic point defects by density functional theory calculations. a Top-view images of the relaxed structure calculated by density functional theory (DFT) of the pristine basal plane and, **b** Se vacancy, **c** WSe_X vacancy, and **d** antisite families of intrinsic defects, respectively. Blue- and dark orange-filled circles stand for W and Se atoms. Light blue and dashed red circles stand for W and Se deficiencies. White arrows show the antisite defects. The lattice constant was calculated at 3.28 Å for the relaxed pristine ML WSe_2.

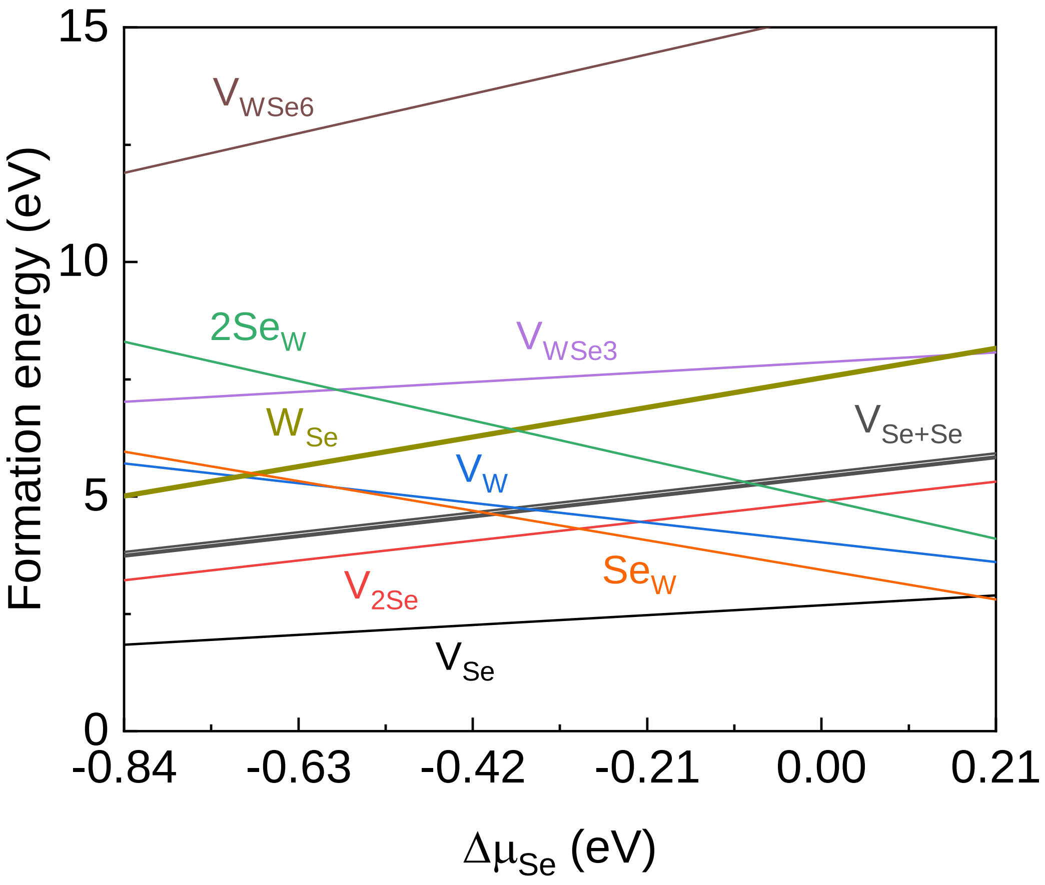

Supplementary Fig. 15 Thermodynamic stability of the various intrinsic defects in the basal plane. Formation energies of different point defects as functions of selenium chemical potential ($\Delta\mu_{Se}$), plotted in the range -0.84 eV < $\Delta\mu_{Se}$ < 0.21 eV. These results are in agreement with the reported values[9].

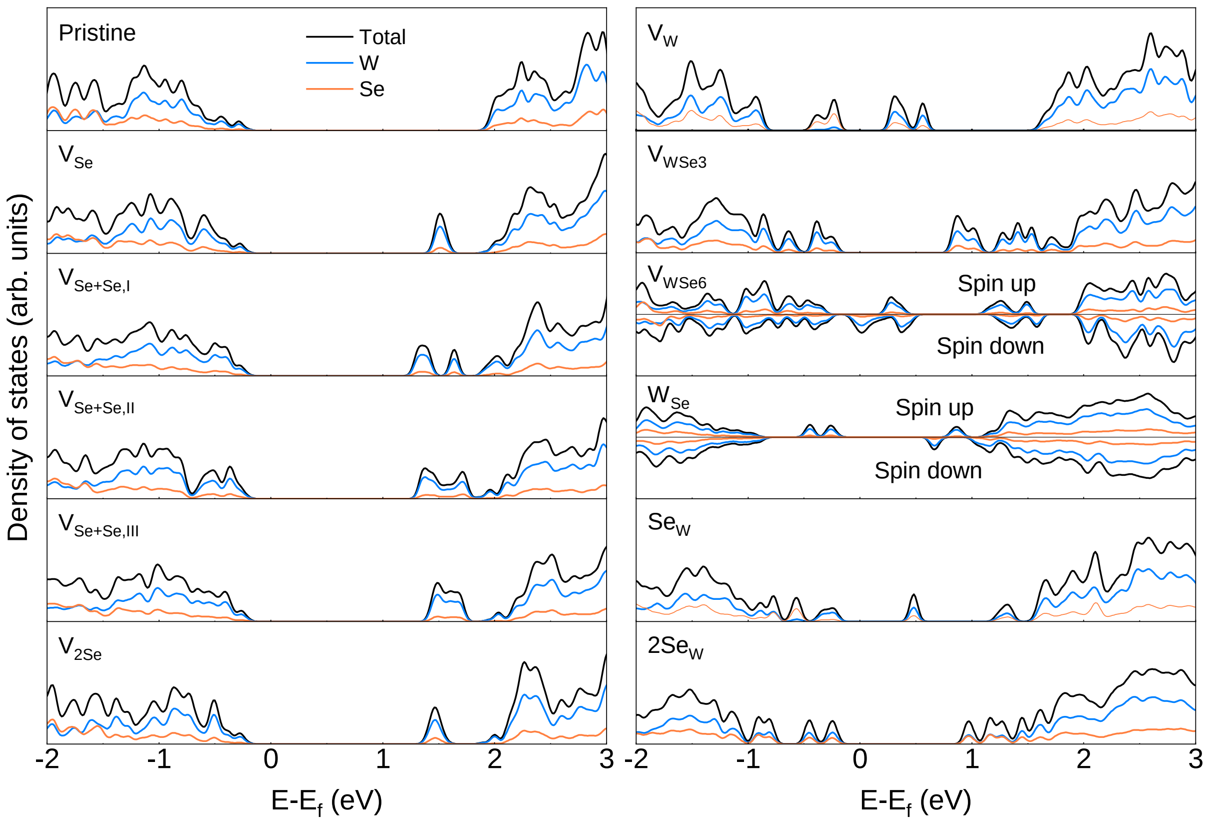

Supplementary Fig. 16 Electronic properties of the basal plane with the intrinsic defects. The HSE06 calculated density of states (DOS) of the pristine basal plane and different intrinsic defects in the basal plane. V_{WSe6} and W_{Se} show a non-zero magnetic moment of 2.00 μB per supercell.

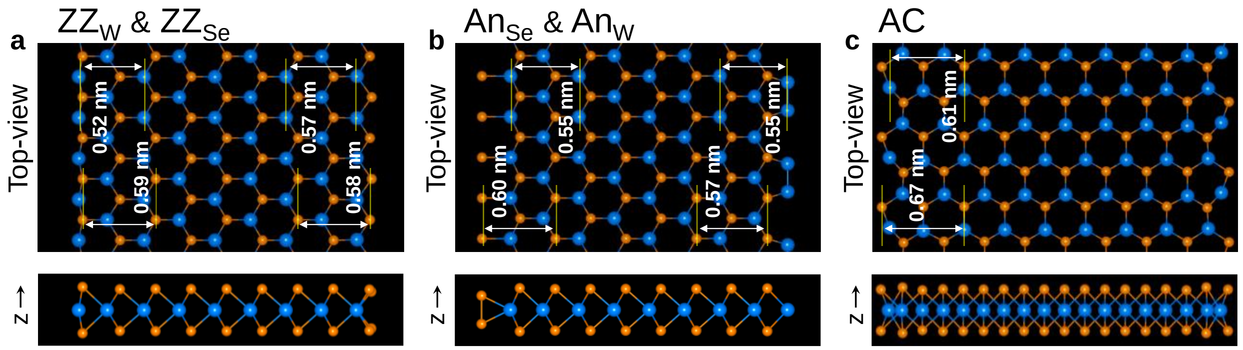

Supplementary Fig. 17 Edge reconstruction by DFT calculation. Top-view and cross-sectional images of the relaxed structures of the edges for different edges: Terminated edge with **a** ZZ_W/ZZ_{Se} (ZZ), **b** An_{Se}/An_W (An), and **c** AC, respectively. ZZ, An, and AC stand for zigzag, antenna, and armchair. Blue- and dark orange-filled circles stand for W and Se atoms. Similarly, An_W/ZZ_W and ZZ_{Se}/An_{Se} configurations were calculated as well (not shown here).

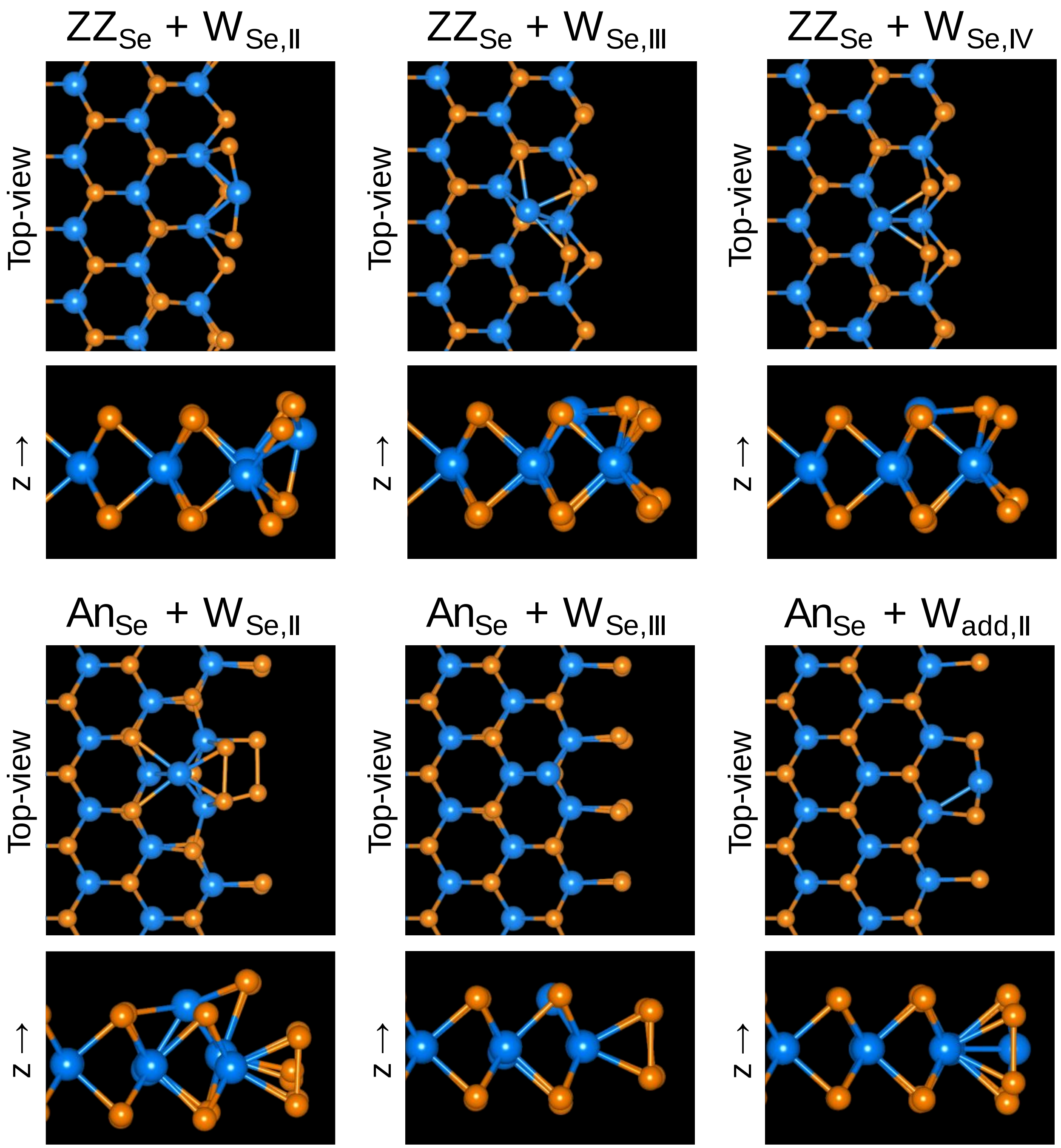

Supplementary Fig. 18 Edge configurations with defects by DFT calculation. Top-view and cross-sectional images of the relaxed structures of the ZZ and An edges with $W_{Se,II}$, $W_{Se,III}$, and $W_{add,II}$ defects. Blue- and dark orange-filled circles stand for W and Se atoms. Notably, V_{Se}, $W_{Se,I}$, and $W_{add,I}$ defects are shown in the Fig. 2c in the manuscript.

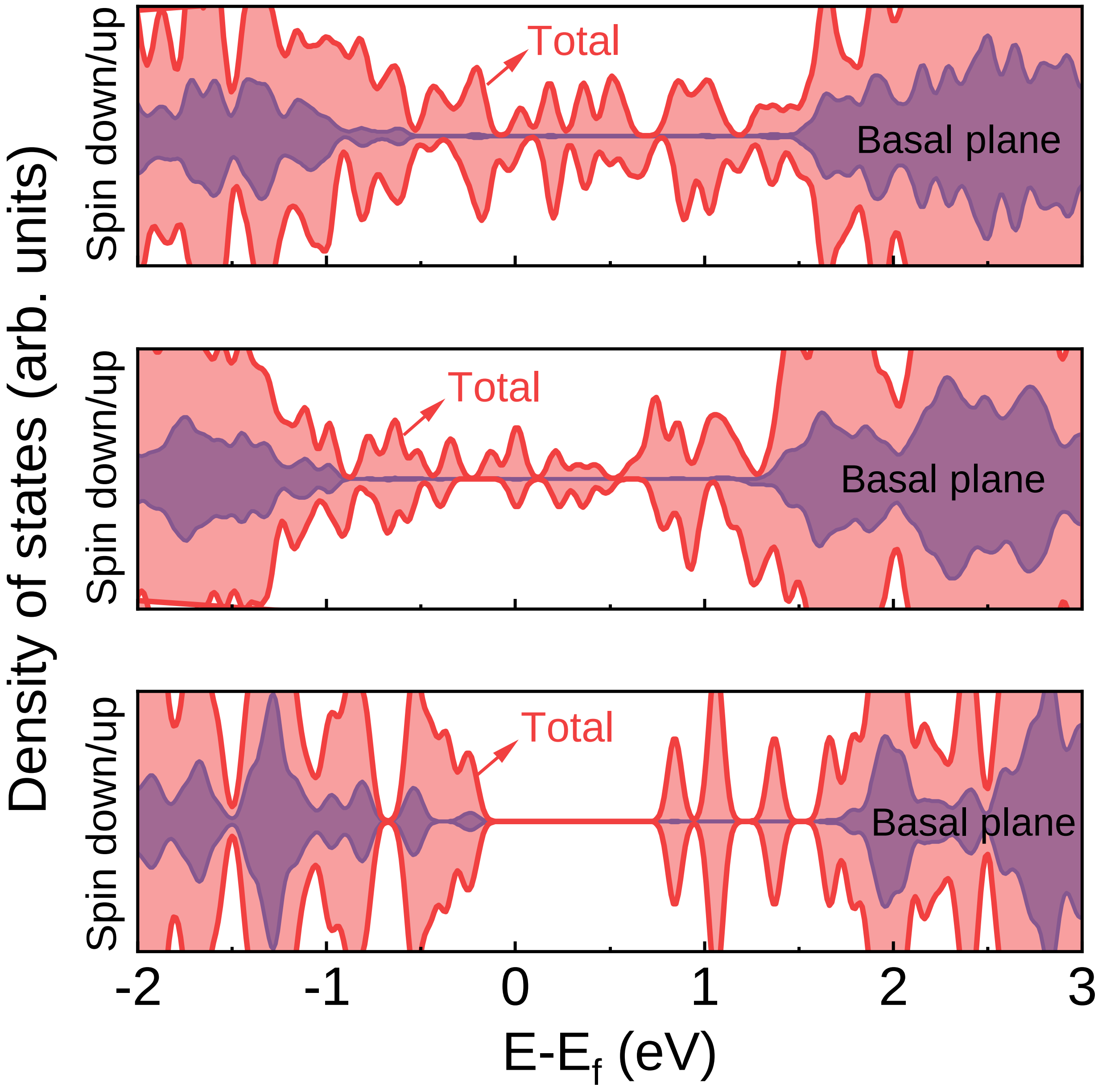

Supplementary Fig. 19 Electronic properties of the reconstructed edges. The HSE06 calculated total local DOS (red line) of the ZZ, An, and AC edges, as shown in Supplementary Fig. 17. The DOSs of the middle regions of the model ribbon are plotted in the background by blue lines.

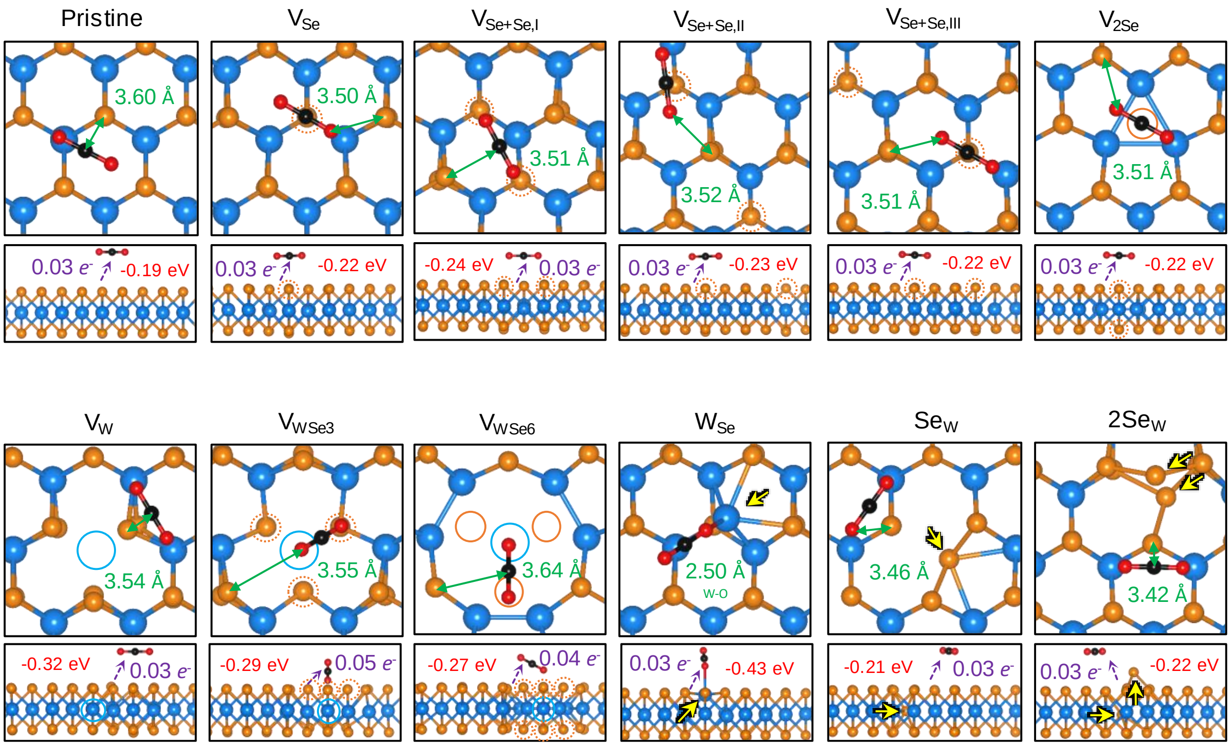

Supplementary Fig. 20 CO_2 adsorption on the basal plane of ML WSe_2. Top and cross-sectional views of the most stable adsorption configurations of CO_2 on the pristine basal plane and intrinsic defects. Charge differences and adsorption energies are added to the panels. Blue- and dark orange- and black- and red-filled circles stand for W, Se, C, and O atoms, respectively. Yellow arrows, dark orange, and blue circles display the antisite defects, V_{Se}, and V_W, respectively. e^- is the magnitude of the negative electric charge carried by a single electron.

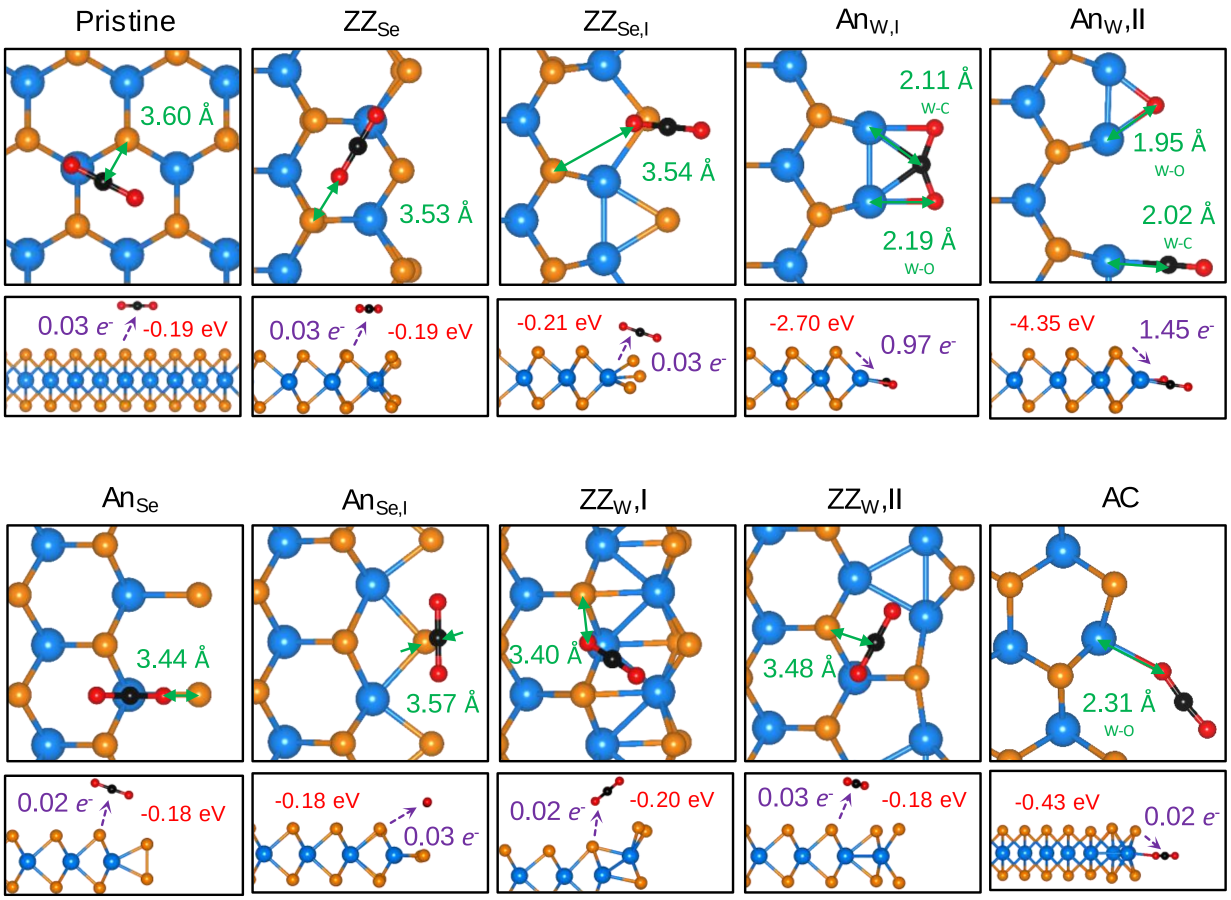

Supplementary Fig. 21 CO_2 adsorption at the regular reconstructed edges of ML WSe_2. Top and cross-sectional views of the most stable adsorption configurations of CO_2 on the pristine basal plane and at the regular reconstructed edges. Charge differences and adsorption energies are added to the panels. Blue- and dark orange- and black- and red-filled circles stand for W, Se, C, and O atoms, respectively. e^- is the magnitude of the negative electric charge carried by a single electron. $ZZ_{Se,I}$ and $An_{Se,I}$ stand for Se-terminated ZZ and An edge with a Se atom at the very edge, respectively.

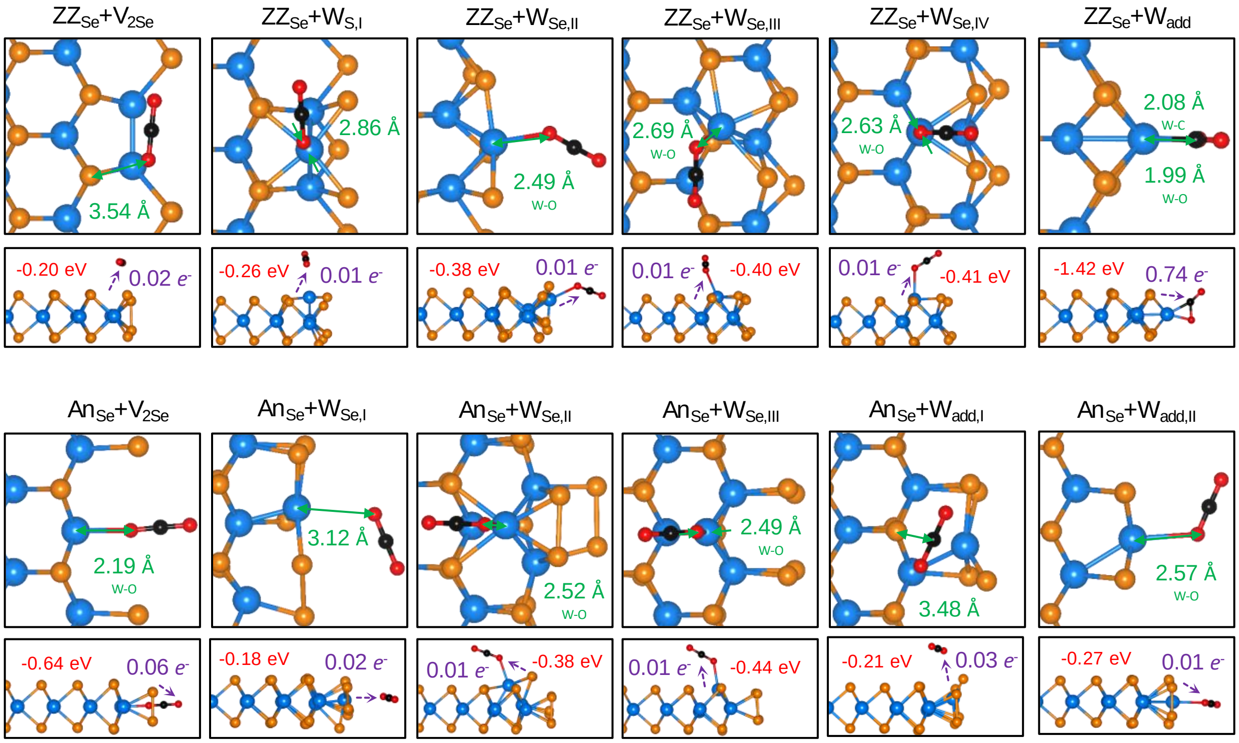

Supplementary Fig. 22 CO_2 adsorption at the defective reconstructed edges of ML WSe_2. Top and cross-sectional views of the most stable adsorption configurations of CO_2 at the defective reconstructed edges. Charge differences and adsorption energies are added to the panels. Blue- and dark orange- and black- and red-filled circles stand for W, Se, C, and O atoms, respectively. e^- is the magnitude of the negative electric charge carried by a single electron.

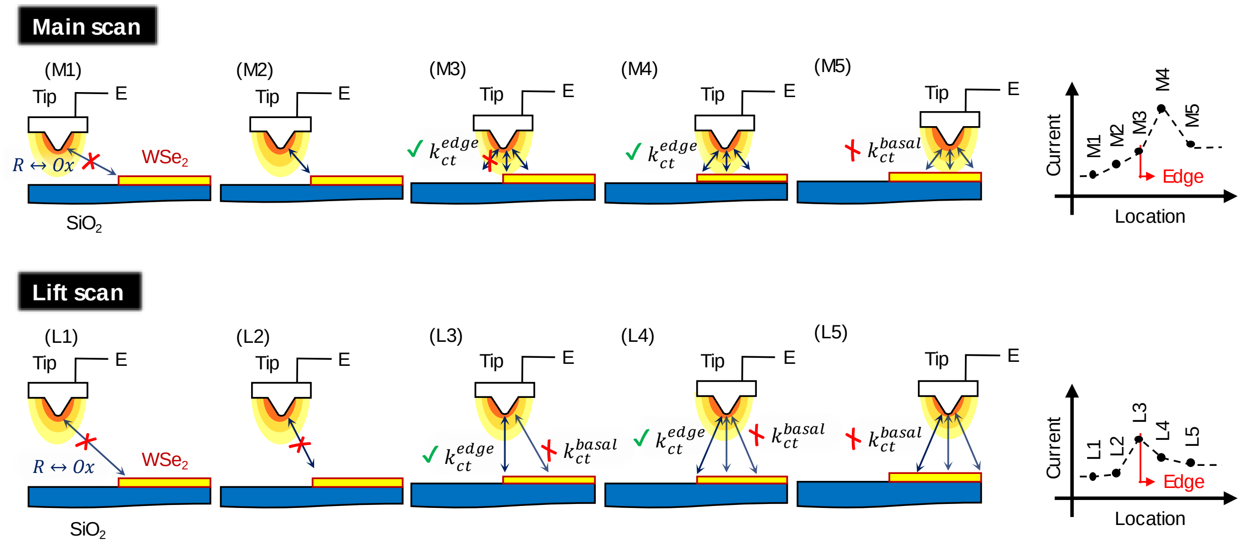

Supplementary Fig. 23 Schematic illustration of AFM-SECM mechanism. Schematic of the main (top panel) and lift (bottom panel) scans. For the main scan: (M1) The tip is far from the ML WSe_2 that measures the feedback current of the insulating SiO_2 substrate. (M2) It is near the edge that can weakly interact with the edge. (M3) It is exactly at the edge with strong interaction. However, there is spatial confinement limiting the feedback signal. (M4) The tip is on the basal plane that can strongly interact with the edge. There is no spatial confinement that results in the maximum feedback current. (M5) It is on the basal where the charge transfer mechanism is not good. For the lift scan: (L1) The tip is far from the ML WSe_2 that measures the feedback current of the insulating SiO_2 substrate. (L2) It is near the edge. It cannot interact with the edge due to its large separation distance. (L3) It is exactly at the edge, at the minimum distance of 100 nm. The tip can interact with the edge results in the maximum feedback current. (L4) It is on the basal plane near the edge that can weakly interact with the edge. (L5) The tip is on the basal where the charge transfer mechanism is not good that is the same as the main scan. E, k_{ct}, and $R \leftrightarrow Ox$ stand for applied electric field, charge transfer, and redox reactions, respectively.

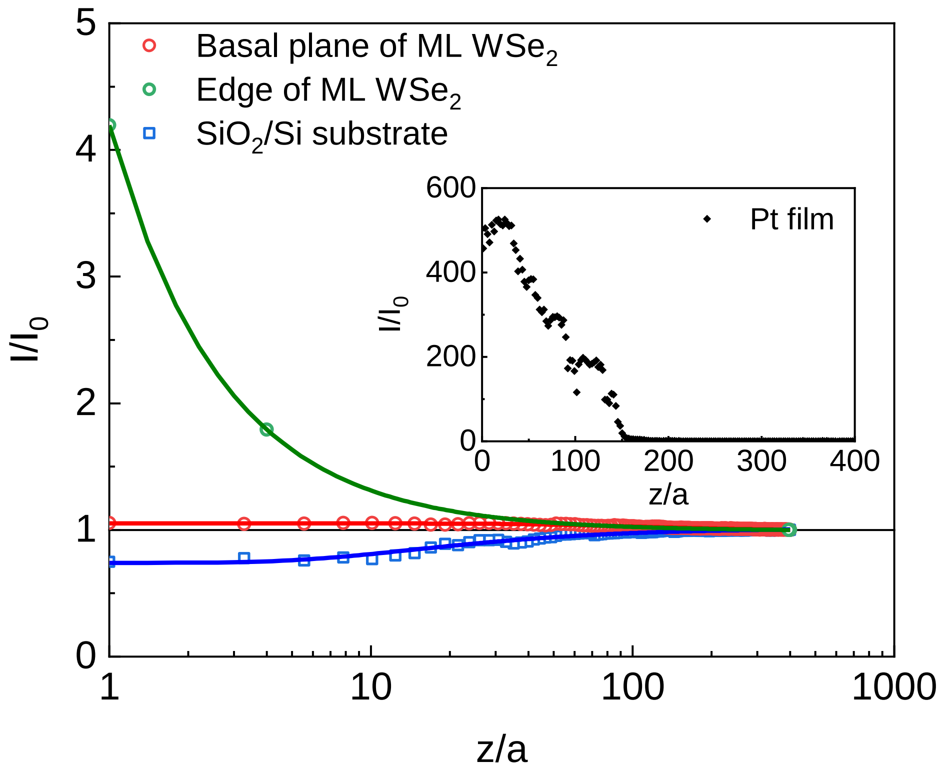

Supplementary Fig. 24 SECM feedback mode. SECM normalized response as a function of the tip–surface distance. Red, green, and blue curves represent the fitted curves for positive ($\frac{I^{+}_{basal}}{I_0}$), positive $\frac{I^{+}_{edge}}{I_0}$ and negative $\frac{I^{-}}{I_0}$ feedback currents, respectively. The inset shows the SECM normalized response of the tip–Pt distance. Our SECM feedback results show that the positive and negative feedback currents are independent of $K^{+}_{2,basal}$ and K^{-}_{2}. So, we have used the following equations to fit the SECM feedback currents: i.e. $K^{+}_{1,basal}$, , K^{-}_{1}, $K^{+}_{3,basal}$, K^{-}_{3}, $K^{+}_{4,basal}$, and K^{-}_{4} of 1.05, 1.35, - 0.063, 0.36, -97.92, -11.48, respectively. Additionally, for the fitted curve $\frac{I^{+}_{edge}}{I_0}$ (positive feedback), $K^{+}_{1,edge}$, $K^{+}_{2,edge}$, $K^{+}_{3,edge}$, and $K^{+}_{4,edge}$ are about 0.3, 3.2, 0.69, and -0.06, respectively.

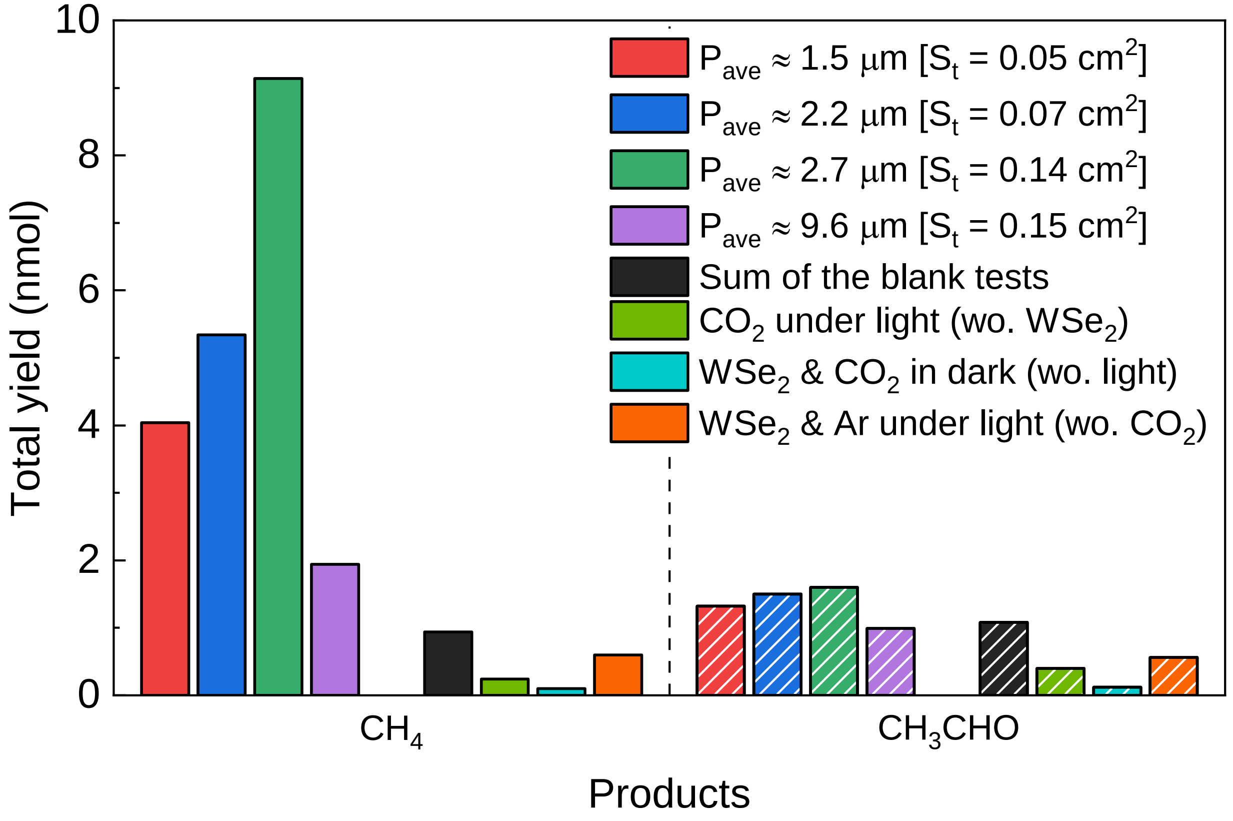

Supplementary Fig. 25 Photocatalyst yields and blank tests. Total yield for methane (CH_4) and acetaldehyde (CH_3CHO) products after 4 h under one sun irradiation. Our results show that acetaldehyde is a minor product because its yield is in the order of the blank tests. Acetaldehyde can be formed from the reduction reaction of impurity as our blank tests show. This side product can consume holes and also form methane. We believe our blank test appropriately subtracts the contribution of the methane from the acetaldehyde side product.

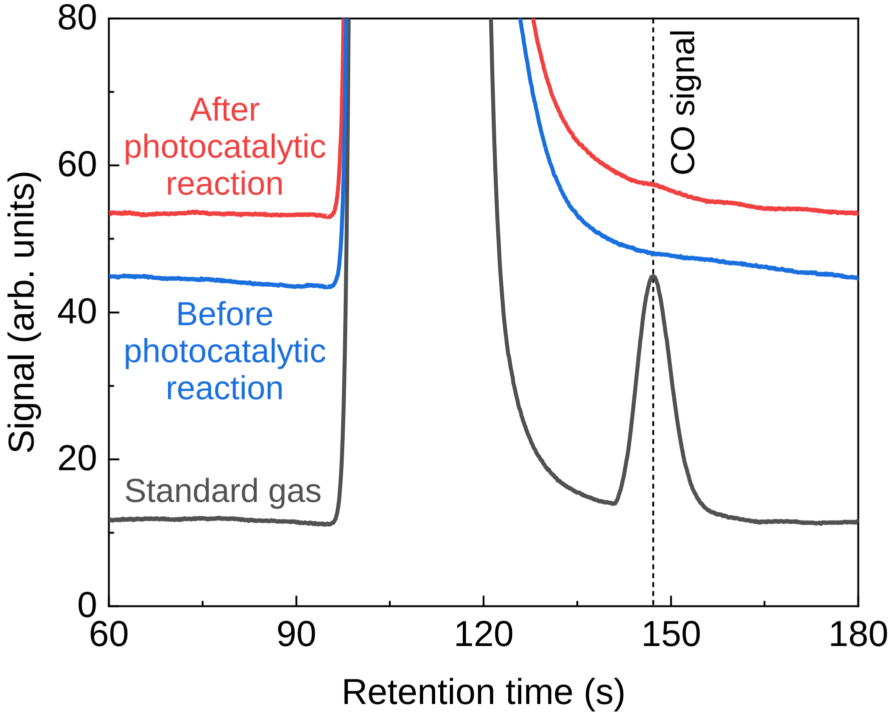

Supplementary Fig. 26 Carbon monoxide yield. Signal versus the retention time for the standard gas (black curve), before photocatalytic reaction (blue curve), and after photocatalytic reaction over ML WSe_2 sample (red line). The inset also shows that CO is not the reduction product.

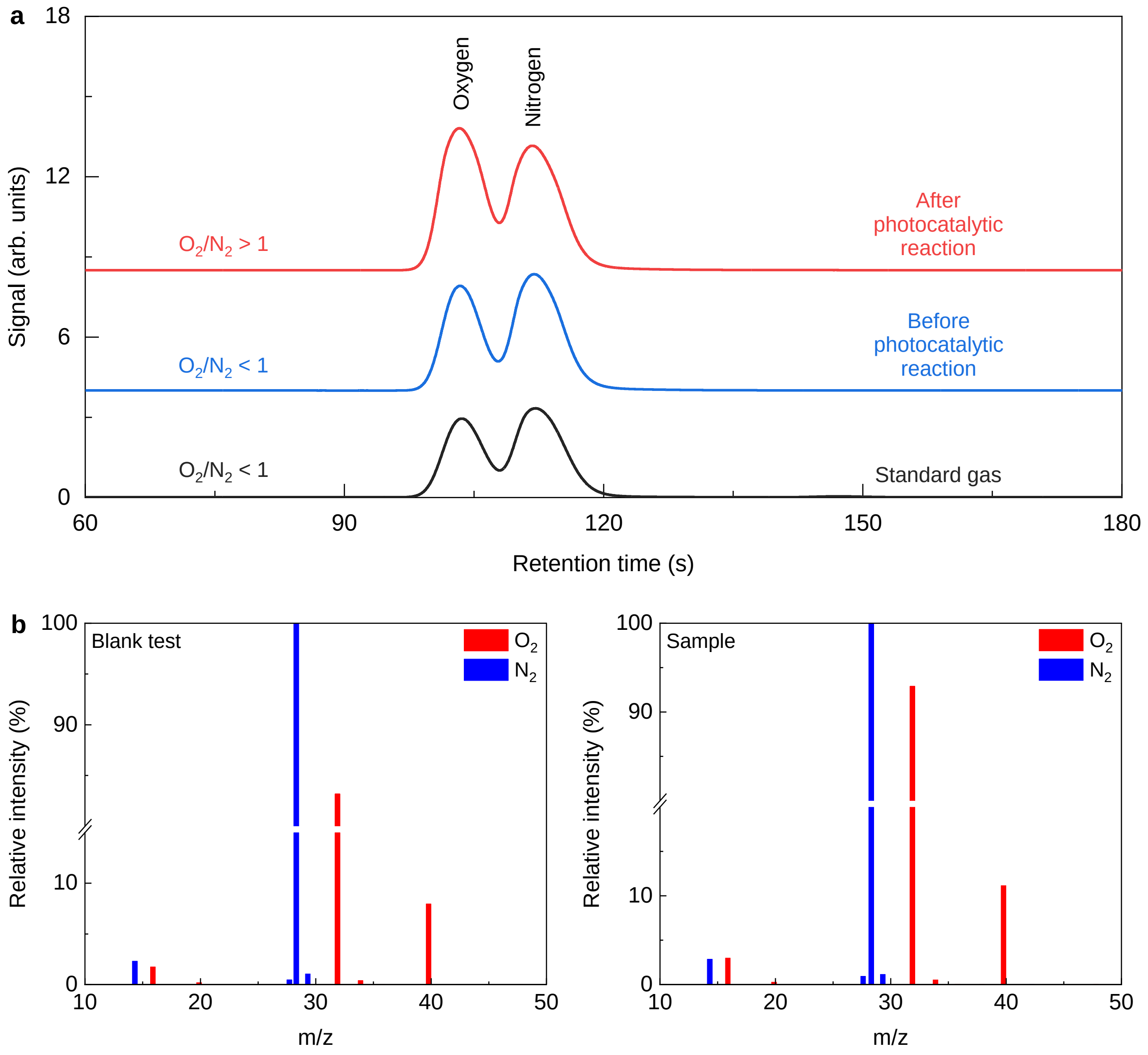

Supplementary Fig. 27 Oxidation product. a O_2 detection from HID for standard gas (black curve), before photocatalytic reaction (blue curve), and after photocatalytic reaction (red curve). **b** O_2 detection from gas chromatography–mass (GC–MS) spectrometry for the blank and sample after the photocatalytic reaction. Both experiments show that the O_2–to–N_2 ratio increased after the photocatalytic reaction.

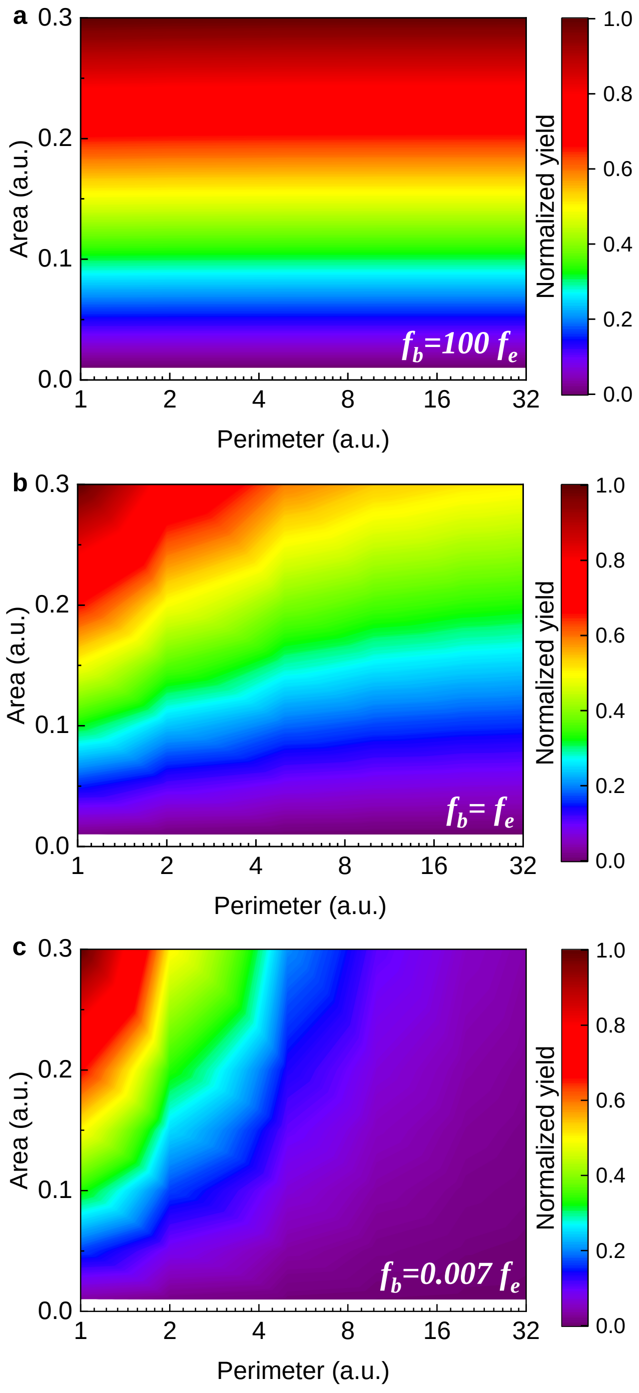

Supplementary Fig. 28 Simulated yield of PC CO_2RR. The simulated color map of normalized yields for **a** $f_b = 100 f_e$ (no contribution from the edge), **b** $f_b = f_e$ (equal contribution), and **c** $f_b = 0.007 f_e$ (experimental data; edge contribution is dominant), respectively.

Supplementary Tables

Supplementary Table 1 Examples of different materials for PC production of CO_2. AQE and IQE stand for apparent and internal quantum efficiencies. CH_4 production rates were calculated based on the geometrical reported area that was exposed to the light irradiation of the samples inside the reactor. It should be noted that the efficiencies presented in two different ways: for overall or for a narrow band (a certain wavelength) of the irradiation spectrum, that we called them "overall" or "at λ nm", respectively. "N/A" and "▲" means not available and data provided by the authors' response.

Year	Co-catalyst @ Catalyst (mass, exposed area)	Light source (Lamp) (Power)	Products	Total gravimetric prod. Rate of C_1 products ($\mu mol\ g^{-1}\ h^{-1}$)	CH_4 prod. rate ($nmol\ cm^{-2}\ h^{-1}$)	Efficiency (CH_4)	Ref.
This work	Monolayer 2H-WSe_2 (~ 0.05–0.27 cm^{-2})	150 W Xenon > 320 nm (0.1 W cm^{-2})	CH_4	N/A	17.5	Maximum IQE 0.23% Overall	–
2022	Amine-functionalized B-doped g-C_3N_4 (15 mg, 4 cm^2)▲	Solar simulator > 420 nm (0.1 W cm^{-2})	CO	~5.48	NA	N/A	10
2020	V_O–TiO_2–Cu_2O (40 mg, 4.9 cm^2)	100 W solar simulator (0.1 W cm^{-2})	CH_4	0.077	0.6	AQE 0.012% at 352 nm	11
2020	Fe–based MOF (2 mg, 2.8 cm^2)	300 W Xenon 400-780 nm (0.4 W cm^{-2})	CO CH_4	~51.8	33.8	AQE ~0.924% Overall	12
2020	C–doped SnS_2 (Thin film, 0.1 cm^2)▲	150 W Halogen (N/A)	CH_4 CH_3CHO	N/A	139.2	AQE ~ 0.09% Overall	13
2020	Pt @ TiO_2–carbon–g–C3N4 (100 mg, 33 cm^2)	300 W Xenon > 420 nm (0.08 W cm^{-2})	CO CH_4	~80.3	~198	AQE ~5.37% at 420 nm	14
2019	Cu & Pt @ blue TiO_2 (40 mg, 4.88 cm^2)	100 W solar simulator (0.1 W cm^{-2})	CH_4 C_2H_6	525	4098.4	AQE 45% at 342 nm	15
2019	CoO_x @ TiO_{2-x} (50 mg, 3 cm^2)	150W UV @ 365 nm (0.02 W cm^{-2})	CO CH_4	1.34	1.5	AQE ~0.0028% at 356 nm	16
2019	Porous BN (30 mg, 9.6 cm^2)	300 W Xenon > 325 nm (5.38 W cm^{-2})	CO	1.17	N/A	N/A	17

2019	Polymer–TiO_2–graphene (20 mg, 3.14 cm^2)	300 W Xenon > 420 nm (0.433 W cm^{-2})	CO CH_4	49.25	175.9	N/A	18
2019	V_s–$CuIn_5S_8$ (5–30 mg, 10 cm^2)	Xenon > 420 nm (0.05 W cm^{-2})	CH_4	6.9 – 8.7	4.4	AQE 0.786% Overall	19
2018	Pt @ Graphene–blue TiO_2 (40 mg, 10 cm^2)	300 W Xenon (0.1 W cm^{-2})	CH_4 C_2H_6	48	N/A	AQE ~5.2% N/A	20
2018	Treated rape pollen (5–30 mg, 2.25 cm^2)	300 W Xenon (0.25 W cm^{-2})	CO CH_4	856.3	70.7	AQE 0.32% at 420 nm	21
2017	Pt @ blue TiO_2 (40 mg, 4.9 cm^2)	100 W Xenon (0.1 W cm^{-2})	CH_4	80.35	655.9	AQE 12.4% at 352 nm	22
2017	Partially oxidized SnS_2 (100 mg, 10 cm^2)	300 W Xenon > 420 nm (0.05 W cm^{-2})	CO	12.28	N/A	N/A	23
2015	CuO–TiO_2 (10 mg, N/A)	40 W Halogen @ 254 nm (0.02 W cm^{-2})	CO CH_4	16.6	N/A	IQE 0.747 at 254 nm	24
2015	CdS–WO_3 (100 mg, N/A)	300 W Xenon > 420 nm (0.15 W cm^{-2})	CH_4	1.02	20.2	IQE 0.4 at 254 nm	25

Supplementary Table 2 Turnover frequency and consumed electron rate. Turnover frequency (TOF) and consumed electron rate (R_e) of the reported works on TMDC materials. Symbols "▲" and "•" stand for wireless and wired systems, respectively. N/A means not available.

Year	Catalyst (System)	Active site	Products (System)	TOF (s^{-1})	N_e	R_e ($e^- s^{-1}$)	Ref.
**This work**	Monolayer 2H-WSe_2	Edge<br>In-plane defects	CH_4	0.48 ± 0.07<br>0.0034 ± 0.0009	8▲	3.8± 0.7<br>0.027 ± 0.004	–
2021	Few-layer MoS_2	S-vacancies	CH_3OH	~0.035 – 0.235	6▲	~0.2 – 1.4	26
2020	1T′ MoS_2<br>1T′ $MoSe_2$<br>1T′ $MoTe_2$<br>1T′ WSe_2	Edge	H_2	3.43<br>0.017<br>0.142<br>0.006	2•	6.86<br>0.034<br>0.284<br>0.012	27
2016	Strained MoS_2 with S-vacancy	S-vacancies	H_2	0.08 – 0.31	2•	0.16 – 0.62	28
2016	WSe_2 nanoflakes	N/A	CO	0.28	2•	0.56	29
2015	Hierarchical MoS_2 nanosheets	N/A	H_2	0.41 – 1.24	2•	0.82 – 2.48	30
2013	Vertically aligned MoS_2 layers	Edge	H_2	0.013<br>0.014	2•	0.026<br>0.028	31
2008	$[Mo_3S_4]^{4+}$ clusters	N/A	H_2	0.07	2•	0.14	32
2007	MoS_2 nanoparticles	Edge	H_2	0.02	2•	0.04	33

Supplementary Notes

Supplementary Note 1. Growth recipe

1–1. Low-pressure vapor deposition. Firstly, 500 sccm Ar gas was introduced by a mass flow controller (Brooksinstrument 5850E) into the quartz tube to flush the gas lines for 10 min. Then, the system was pumped down to a vacuum of < 0.01 Torr and kept for 15 min. After that, the desired flow of Ar gas, from 30 to 70 sccm, was used as the carrier gas for 10 min. The pressure inside the quartz tube was controlled in the range of 0.40 to 0.50 Torr by a mechanical valve. Finally, the furnace center was heated up to 950 °C (with a rate of 31 °C min^{-1}) and kept for 5 min before it was cooled to 300 °C naturally, followed by a fan-assisted fast cooling to room temperature. Supplementary Note Fig. 1 illustrates the detailed growth recipe of the growth.

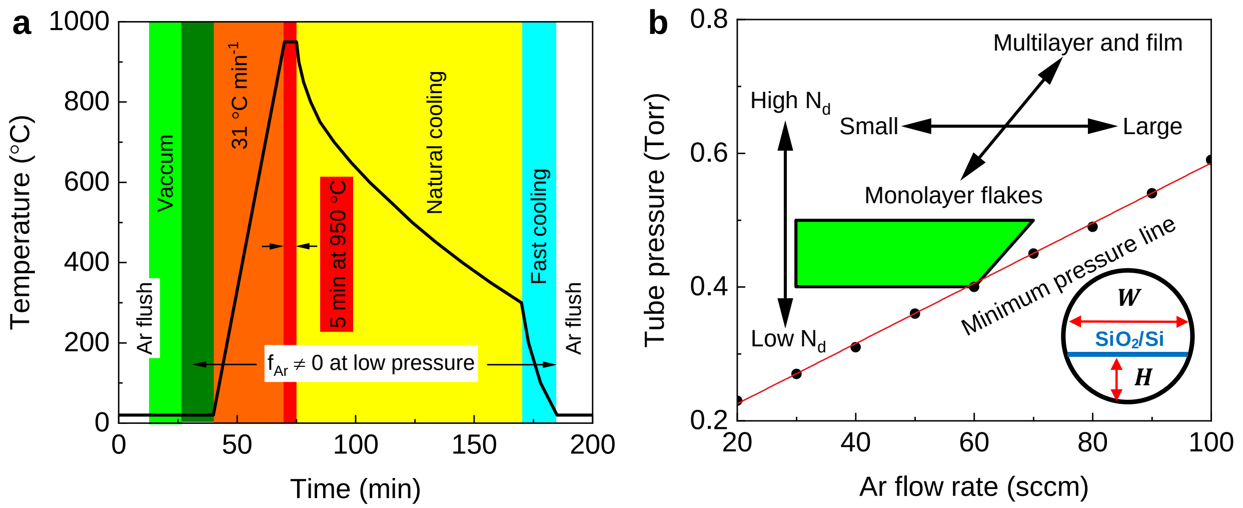

Supplementary Note Fig. 1 Illustration of the growth recipe of low-pressure vapor deposition. a Temperature control curve of alumina crucible during the growth process and **b** pressure versus Ar flow rate diagram for optimized growth, respectively. The green region in **b** shows the optimized parameters to grow uniform and well-separated monolayer WSe_2 flakes with a perimeter in the range of 1.5 to 25 μm. Black-filled circles and the fitted red line illustrate the minimum accessible pressure of the system. Moreover, it presents how the tube pressure and Ar flow rate can vary nucleation density (N_d), size, and thickness of the flakes. We observed that the size and N_d can be controlled by varying the height of the substrate (see the inset of **b**).

We performed several experiments and analyses — such as photoluminescence (PL), atomic force microscopy (AFM), high-resolution transmission electron microscopy (HRTEM), and selected area electron diffraction (SAED) patterns — to figure out the number of layers of the grown WSe_2 flakes. Monolayer (ML) WSe_2 has an intense PL intensity which is much stronger (about one order of magnitude) than the bilayer (BL) flake (Supplementary Note Fig. 2).[34] Moreover, the BL flake (with a thickness of ~1.5 nm) has an obvious smaller optical band gap of ~1.62 eV (with a red-shift of ~20 nm) as compared with the ML one.

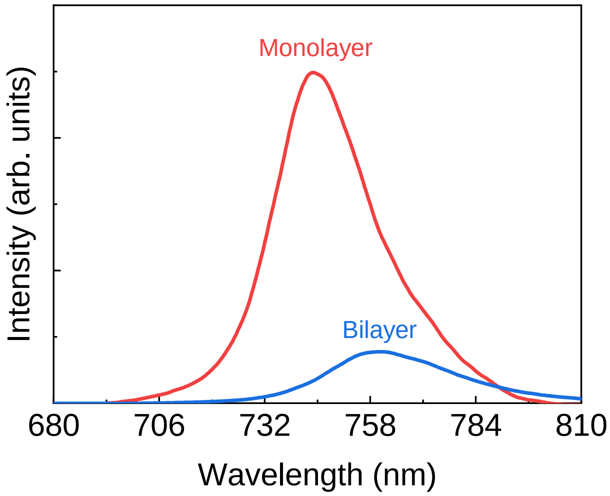

Supplementary Note Fig. 2 Photoluminescence spectra of the ML and BL WSe_2. The excitation wavelength is 473 nm (blue laser).

Then, we have used PL measurement to check the uniformity of the number of layers after the growth process. Supplementary Note Fig. 3 presents the PL spectra of the various flakes on different samples. All these spectra show the optical band gap of ~1.68 eV, with an error of $\leq$ 0.01 eV. Regarding their intense intensity and high band gap as compared with BL flakes, one can conclude that the number of layers is uniform (i.e., ML) in each sample.

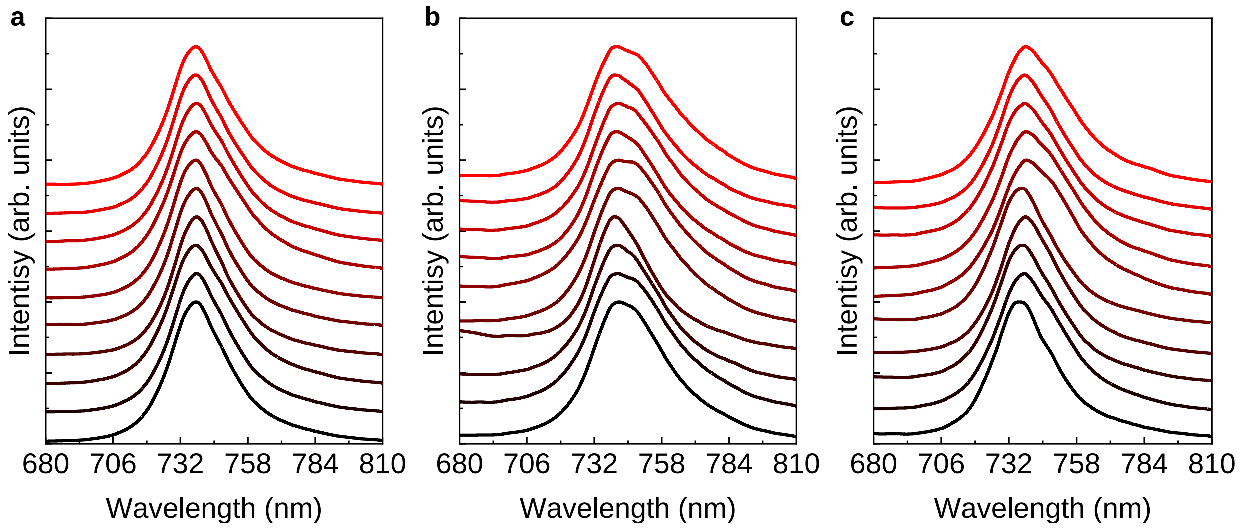

Supplementary Note Fig. 3 Uniformity of the number of layers by photoluminescence. The PL spectra of the various flakes at different random places in each sample with average flake sizes of **a** $L \approx 0.7$ μm, **b** $L \approx 1.8$ μm, and **c** $L \approx 4.2$ μm. Notably, the variation of the PL intensities is less than 20% for each panel.

Notably, we have observed the presence of some BL regions on some of the large flakes mostly close to the nucleation sites (Supplementary Note Fig. 4a). Our investigations show that rather than initial nucleation sites, secondary nucleation sites can also be the origin of the growth of the second layers. AFM and HRTEM images show that the apexes of some of the flakes are the secondary nucleation sites (Supplementary Note Fig. 4b and c). Notably, the density of the BL region is much lower in the smaller flakes due to the less feeding rate during the growth process. Supplementary Note Fig. 4d and e reveal that the second layer is already twisted to a small angle due to an energetically favorable stacking structure, displaying moiré patterns. So, the ensemble averaging estimates a few percent of BL flakes in each sample.

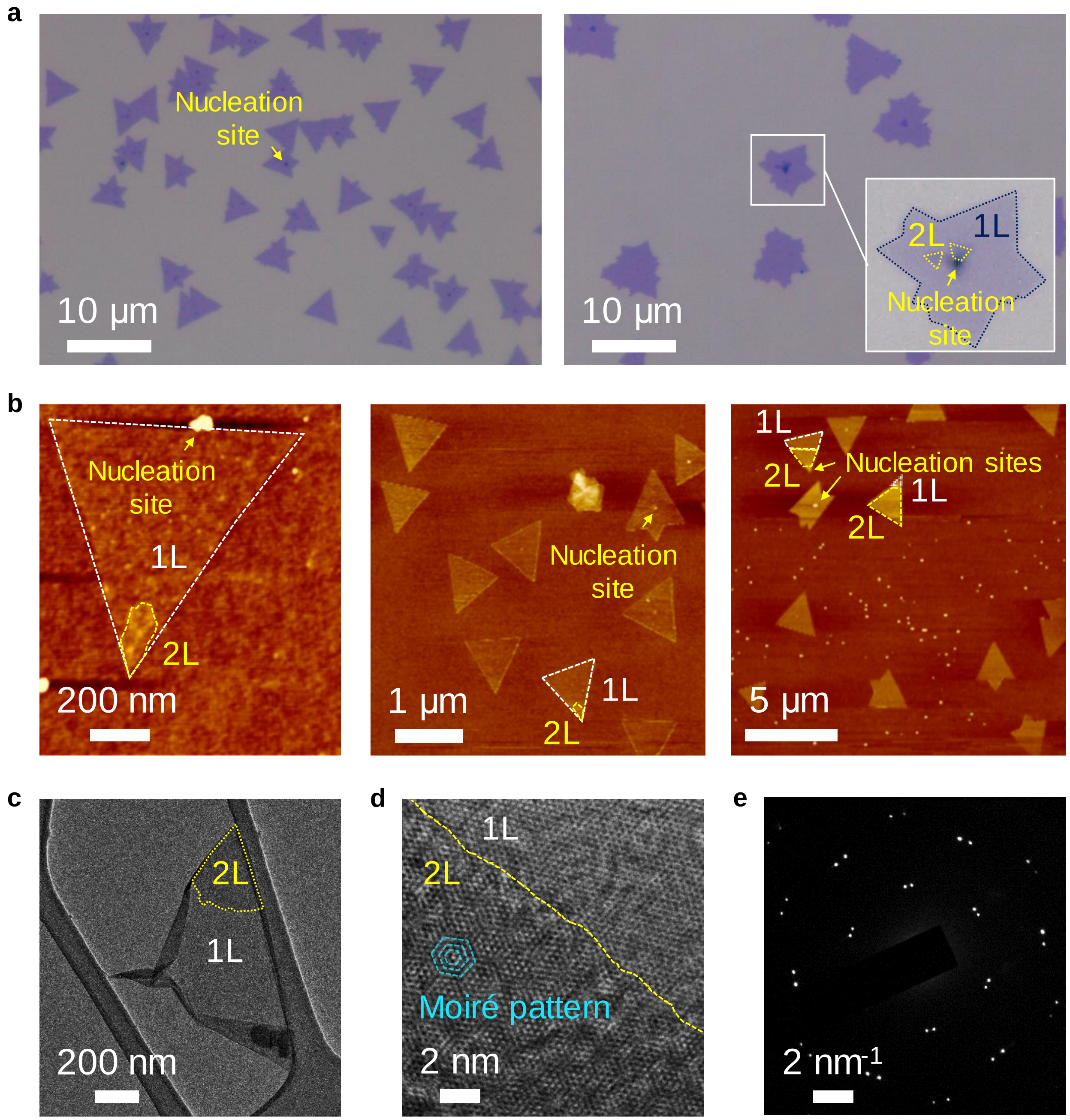

Supplementary Note Fig. 4 Growth of the partial second layer. a Optical microscope images of flakes. It should be noted that the ML and BL regions cab be distinguished quickly by their different contrasts **b** AFM height profiles. **c** TEM and **d** HRTEM images. **e** SAED pattern of the BL region.

1–2. Atmospheric-pressure chemical vapor deposition. Two quartz boats containing WO_3 powder (50 mg) and Se powder (10 mg) were placed at the center and a distance of 17.5 cm upstream from the center of the furnace, respectively. A SiO_2(300 nm)/Si substrate was placed

facing up at a distance of 7.5 cm downstream from the center of the WO_3 source. Before the growth, the system was flushed with 100 sccm Ar for 20 min Finally, the center of the furnace was heated up to 925 °C (with the rate of 30 °C min^{-1}) and kept for 5 min before a slow cooling to 800 °C in 1 h, followed by a naturally cooling to room temperature. Notably, both Ar and H_2, with a flow rate of 50 and 2.5 sccm, respectively, were introduced as the carrier gases during the chemical vapor deposition process.

Supplementary Note 2. Monolayer WSe_2 transfer

The top side of the $WSe_2/SiO_x/Si$ surface was spin-coated (3000 rpm for 20 s) with 5 wt% PMMA (M_w ~120,000; Sigma–Aldrich 9011-14-7) in chlorobenzene solvent (99.9%; Sigma–Aldrich 108-90-7) and baked at 140 °C for 15 min. Then, it was immersed into 1 M KOH until the film peels off from the substrate and floats on the etchant surface. The PMMA/WSe_2 film was washed with deionized (DI) water several times and then transferred onto a prepared substrate (carbon-coated copper grid and glass). After drying at 75 °C for 30 min to enhance the adhesion, finally, the PMMA supporting layer was dissolved by acetone and isopropyl alcohol.

Supplementary Note 3. Characterizations

3–1. Analyzing optical microscopy images. Each substrate was partitioned into similar boxes by considering an imaginary 3 × 3 grid to calculate the average lateral size, perimeter, and area of the flakes. Then, an image, with a magnification of 100×, was recorded from the center of each box. Given that, the average values, using ImageJ software (version 1.53a), were computed by measuring the lateral size, perimeter, and area of several hundred flakes from all recorded images. Nucleation density was measured by counting the number of micro-scale flakes over the recorded image directly. A similar method, by recording 56 images, with a magnification of 100×, from an imaginary 7 × 8 grid, was applied to measure the total area of the transferred film onto the glass substrate.

3–2. Fitting X-ray photoelectron spectroscopy spectra. X-ray photoelectron spectroscopy (XPS) technique was used to find the x value with an Al–K_α X-ray source at the energy of 1486.6 eV. Fixing the C 1s peak to 284.6 eV resulted in the calibration of binding energies. CasaXPS software (version 2.3.20), with a Gaussian–Lorentzian product function and an error of ± 0.05 eV, was used to fit the XPS spectrum window. Regarding the X-ray Sources at 54.7°, atomic sensitivity factors (ASFs) considered for Se 3d and W 4f were 0.853 and 3.523, respectively.

3–3. Photoluminescence and Raman experiments. Photoluminescence (PL) and Raman spectra were collected on confocal Raman systems using continuous wave (CW) 473 with laser power ~20 mW, 632 nm with laser power ~15 mW (NTEGRA Spectra; NT–MDT), 532 nm with laser power from about 0.5 to 15 mW, and picosecond pulse diode 405 nm (HORIBA; iHR550) lasers. The beam diameter of the lasers was about 1.5 μm. Power-dependent PL experiments were carried out by passing the beam of 405 nm laser, with laser power from about 0.5 to 15 mW, through a filter before the sample. A cryogenic stage (Linkam BCS196) equipped with the 405 nm laser, under liquid N_2 (Linkam LNP96 cooling system), was used to perform the low-temperature experiments. Time-resolved PL (TRPL) measurements were carried out using Hamamatsu time-correlated single-photon counting (TCSPC; HORIBA Jobin Yvon iHR550), equipped with an H10330A–75 NIR–PMT detector (HORIBA Jobin Yvon iHR 320), and violet laser with duration and repetition frequency of 60 ps and 40 MHz, respectively. We used the following mono-exponential decay function to fit the TRPL curves:

$$\begin{cases} I(t) = A_0 + A \times H(t - t_0) \times e^{\frac{t-t_0}{\tau}} \\ H(t - t_0) = \frac{1}{2}\left[1 + \text{erf}\left(\frac{t-t_0-\frac{\sigma^2}{\tau}}{\sigma\sqrt{2}}\right)\right] e^{\frac{\sigma^2}{2\tau^2}}, \end{cases} \tag{1}$$

where A_0, A, $H(t - t_0)$, t, t_0, σ, and τ are constant offset, normalized radiative recombination coefficient, Heaviside step function, time, starting decay time, standard deviation account for the instrument response function (IRF), and recombination time scale[35]. In our system, t_0 and σ are 3.945 and 0.045 s, respectively.

3–4. Overall absorption percentage of ML WSe_2. The Beer–Lambert law was the law used to estimate the overall absorption percentage ($\beta_{AM\ 1.5G}$) by the irradiation of an AM 1.5G Xe lamp using the following equation for a film,

$$\beta_{AM\ 1.5G} = \frac{\int [1 - T(\lambda < \lambda_g)]\phi(\lambda) d\lambda}{\int \phi(\lambda) d\lambda}, \tag{2}$$

where λ [nm], $T(\lambda < \lambda_g)$, and $\phi(\lambda)$ [s^{-1} cm^{-2}] are wavelength, transmittance, and incident photon flux, respectively[36]. We considered the photon energy that can produce electron–hole pair by the energy larger than the bandgap of the semiconductor, i.e. $\lambda < \lambda_g$. The transmittance of the transferred film on the transparent substrate is a function of the coverage-dependent absorbance, $A_\theta(\lambda)$,

$$T_{\theta<1}(\lambda) = 10^{-A_\theta(\lambda)}, \tag{3}$$

where θ is the coverage, i.e. $\theta = S_f/S_s$ there, S_f and S_s are the area of the transferred film and optical slit of the apparatus, respectively. The absorbance of the transferred film can be given by,

$$A_\theta(\lambda) = \theta\alpha d = \theta A(\lambda), \tag{4}$$

where α, d = 8 Å (measure by atomic force microscope height profile), and $A(\lambda)$ are the absorption coefficient, the thickness of ML flakes, and coverage-independent absorbance, respectively. Therefore, the corrected transmittance can be calculated by the following equation,

$$T(\lambda) = 10^{-\frac{A_\theta(\lambda)}{\theta}}. \tag{5}$$

It shows that the average coverage-corrected absorption, i.e. $1 - 10^{-\frac{A_\theta(\lambda)}{\theta}}$, is about 7–8% in the visible region illustrating a strong light–matter interaction by the ML WSe_2 that is in agreement with the theoretical prediction[37]. By substituting the Eq. (S5) in Eq. (S2), the

$$\beta_{AM\ 1.5G} = \frac{\int [1-10^{-\frac{A_\theta(\lambda<\lambda_g)}{\theta}}]\phi(\lambda)d\lambda}{\int \phi(\lambda)d\lambda}. \tag{6}$$

By considering the spectrum of the utilized Xe lamp (see Fig. 1e in the manuscript) and λ_g = 740 nm, the average $\beta_{AM\ 1.5G}$ is estimated at 4.4 ± 0.3 % for different S_f in the range of 0.08 to 0.10 cm^2.

Supplementary Note 4. Density functional theory calculations

4–1. Computational method. Spin-polarized density functional theory (DFT) calculations were performed using the Vienna ab initio simulation package (VASP)[38]. The ion-electron interaction was described with the projector augmented wave (PAW) method[39], and the electron exchange-correlation functional was calculated using the generalized gradient approximation (GGA) with Perdew–Burke–Ernzerhof (PBE)[40]. The electron wave functions were expanded on a plane-wave basis set with an energy cutoff of 500 eV. For considering the van der Waals (vdW) interaction, the dispersion-correction DFT–D3 method[41] was added to the PBE functional, and the calculated lattice constants (a = b = 3.286 Å, and c = 12.995 Å) of the 2H-WSe_2 unit cell were in good agreement with experimental data (a = b = 3.282 Å, and c = 12.96 Å)[42]. A conjugate gradient method was applied to relax the geometry until the residual force on each atom was smaller than 0.01 eV $Å^{-1}$, and the criterion for converging total electronic energies was less than 10^{-5} eV.

For the DOS calculations, since the GGA is well-known for underestimating band gaps of semiconductors, we used the HSE06 hybrid functional[43] to perform the static electronic calculation based on the optimized structure at the PBE level. Due to the high demand for the HSE06 calculation, the energy cutoff was set to 400 eV, and Γ-centered k-point meshes of 6 × 6 × 6 and 3 × 1 × 1 were adopted to calculate the DOS for the monolayer and nanoribbon models, respectively.

4–2. Models of basal plane. The basal plane of ML WSe_2 nanoflakes was simulated by using a 4 × 4 monolayer model. Various in-plane defects observed by the experiment were also calculated, resulting in pristine and eleven defective monolayers (Supplementary Fig. 14): ML with a Se-vacancy (V_{Se}), ML with two Se-vacancy in different arrangements, ML with one W-vacancy (V_W), ML with WSe_3 vacancy (V_{WSe3}), ML with WSe_6 vacancy (V_{WSe6}), ML with a W atom replacing one Se atom (W_{Se} antisite defect), ML with one Se atom replacing one W atom (Se_W antisite defect), and ML with two Se atoms replacing one W atom ($2Se_W$ antisite defect). It should be noted that the symmetry breaking results of the W_{se}, Se_W and $2Se_W$ antisites are about 0.10, 0.66, and 0.66 eV (more stable than the symmetric results which have a C_{3v} symmetry on the defect sites), respectively.

4–3. Models of regular edges. In this study, three types of regular edges, namely zigzag (ZZ), antenna (An), and armchair (AC) edges terminated with Se and W were investigated. 4 × 4 nanoribbon models (48 atoms) were used to calculate the relaxed configurations. Moreover, twelve atoms of the other side of the edge were fixed in the positions of the perfect monolayer. Supplementary Note Fig. 5 shows the optimized results of the regular edges were sensitive to the initio structures and the calculation setting for treating symmetry — for VASP code, ISYM = 2 or 0 was set to switch on or off the use of symmetry — especially for the W-terminated edges. E.g., the optimized result of $ZZ_{W,symmetric}$ can be obtained from an ordered initio structure with a C_h point group symmetry and with the calculation setting of ISYM = 2. When the calculation setting of ISYM = 0 was set, the optimized result is the most reconstructed $ZZ_{W,I}$ edge. $ZZ_{W,II}$ edge was also achieved from a disordered initio structure. These two reconstructed ZZ_W edges are energetically stable than the $ZZ_{W,symmetric}$. Besides, similar structures of $ZZ_{W,I}$ and $ZZ_{W,II}$ have been calculated in other DFT studies for reconstructed MoS_2[44] and transition metal dichalcogenides (TMDCs)[45] edges. It should be noted when the CO_2 molecule was introduced to the $ZZ_{W,symmetric}$ configuration,

it was observed that $ZZ_{W,symmetric}$ reconstructed into the $ZZ_{W,I}$ or $ZZ_{W,II}$ configurations due to the symmetry breaking.

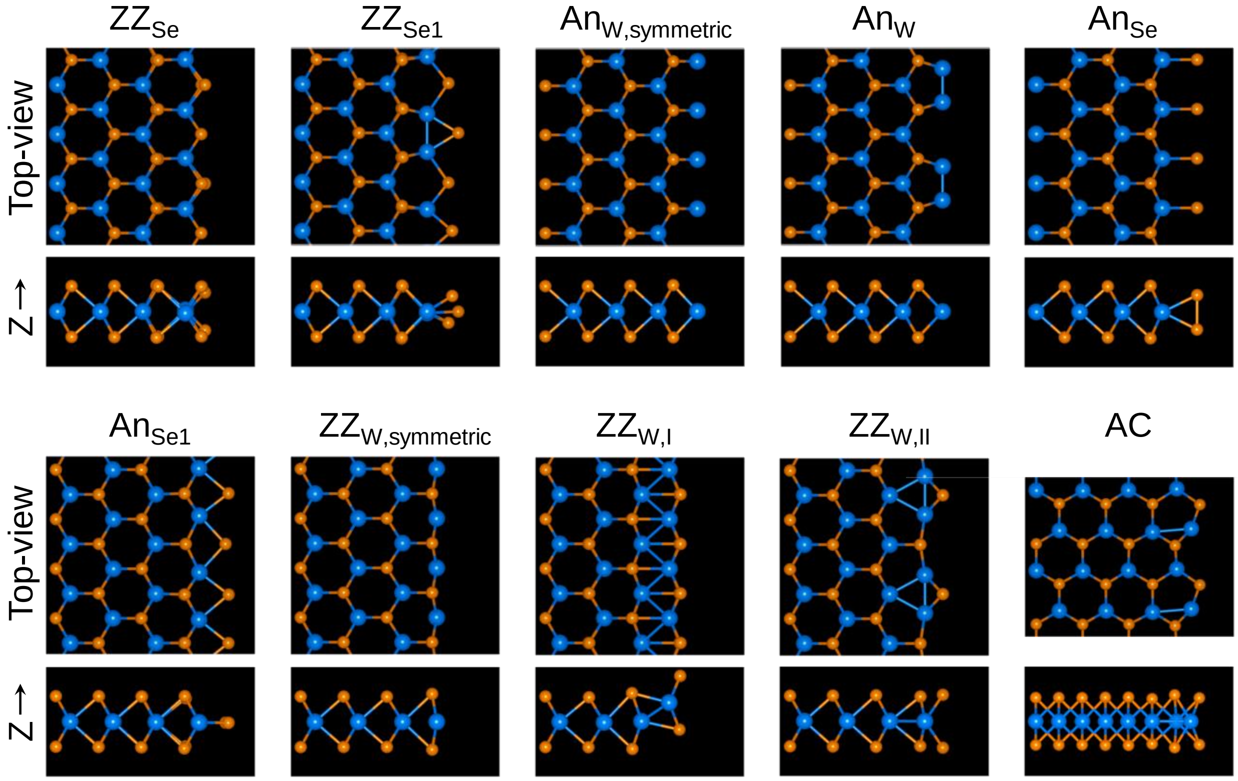

Supplementary Note Fig. 5 Regular edge by DFT calculation. Top-view and cross-sectional images of the relaxed structures of the edges for different edges: Terminated edge with ZZ_W/ZZ_{Se} (ZZ_{Se1}), An_{Se}/$An_{W,symmetric}$ (An_W), An_w/An_{Se}, ZZ_{se}/An_{Se1} ($ZZ_{W,symmetric}$, $ZZ_{W,I}$, $ZZ_{W,II}$), and AC, left to right. ZZ, An, and AC stand for zigzag, antenna, and armchair. Blue- and dark orange-filled circles stand for W and Se atoms. The outmost eight Se atoms and four W atoms of the left edge were fixed in each model.

4–4. Models of defective edges. There can be a large number of defective edges. However, we focused on specific defects occurring at the ZZ_{se} and An_{se} edges due to a Se-rich condition of our grown WSe_2 nanoflakes (Fig. 1d in the manuscript). Notably, a W-terminated edge can already adsorb CO_2. So, our focus in this calculation was on the defects at the Se-terminated edge. As shown in Fig. 3 in the manuscript, we proved that the stronger interaction between WSe_2 nanoflakes and CO_2 molecules is via the bonding of exposed W atoms and CO_2. Due to this reason, V_{2Se}, W_{se}, and W_{add} (W adatom to the edges) defects that can provide exposed W atoms were

introduced to the ZZ_{Se} and An_{Se} edges for studying the CO_2 adsorption (Supplementary Note Table 1). We did not study the defect at the AC edge because it already can adsorb CO_2.

Supplementary Note Table 1 Models of defective edges. ZZ_{Se} and An_{Se} initial input configurations with different defects are shown and explained.

Structure	Explanation	Figure
**ZZ_{Se}**	Input file	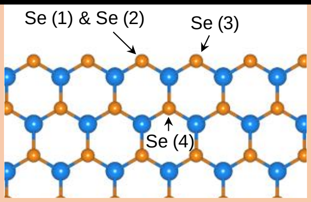
**ZZ_{Se} + V_{2Se}**	Se (1) and Se (2) are removed	Fig. 2c (Manuscript)
**ZZ_{Se} + W_{Se}**	Se (1) replaced by W<br>(relaxed configuration I)	Fig. 2c (Manuscript)
**ZZ_{Se} + W_{add}**	W is added between Se (1) and Se (3)	Fig. 2c (Manuscript)
**ZZ_{Se} + $W_{Se,II}$**	Se (1) replace by W<br>(relaxed configuration II)	Supplementary Fig. 18
**ZZ_{Se} + $W_{Se,III}$**	Se (4) replaced by W<br>(asymmetric relaxed structure)	Supplementary Fig. 18
**ZZ_{Se} + $W_{Se,IV}$**	Se (4) replaced by W<br>(symmetric relaxed structure)	Supplementary Fig. 18
**An_{Se}**	Input file	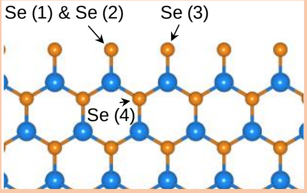
**An + V_{2Se}**	Se (1) and Se (2) are removed	Fig. 2c (Manuscript)
**An + W_{Se}**	Se (1) replaced by W	Fig. 2c (Manuscript)
**An + W_{add}**	W is added between Se (1) and Se (3)<br>(relaxed configuration I)	Fig. 2c (Manuscript)
**An + $W_{Se,II}$**	Se (4) replaced by W<br>(relaxed configuration I)	Supplementary Fig. 18
**An + $W_{Se,III}$**	Se (4) replaced by W<br>(relaxed configuration I)	Supplementary Fig. 18
**An + $W_{add,II}$**	W is added between Se (1) and Se (3)<br>(relaxed configuration II)	Supplementary Fig. 18

4–5. CO_2 adsorption calculation. To find the most stable CO_2 adsorption results, various initial configurations of a CO_2 molecule were considered to cover the different sites of the basal plane and edge models. Before optimization, the introduced CO_2 molecule was located more than 3.2 Å far from the atoms of optimized WSe_2 models with the CO bonds of 1.177 Å and an angle of 180°. The binding energy ($E_b^{CO_2}$) is defined as:

$$E_b^{CO_2} = E_{total} - E_{WSe_2} - E_{CO_2} \quad (7)$$

where E_{total} is the calculated total energy of the WSe_2 monolayer or nanoribbon with a CO_2 molecule adsorbed on it. E_{WSe_2} is the total energy of the isolated WSe_2 monolayer or nanoribbon, and E_{CO_2} is the total energy of an isolated CO_2 molecule. The amount of charge transfer (charge difference δq) between the CO_2 molecule and adsorbent was evaluated by using the Bader charge analysis[46].

4–6. Calculation of formation energy. The defect formation energies were calculated by[47],

$$\Delta\varepsilon_f = E_{defect} - E_{pristine} - \sum_i n_i \mu_i, \quad (8)$$

where E_{defect} and $E_{pristine}$ are the total energies of the supercell with specific defects and the related pristine system (the perfect monolayer or the corresponding nanoribbon without defects), respectively. n_i is the number of atoms i (for i = W and Se) being added to (> 0) or removed from (< 0) the pristine system when creating specific defects, and μ_i is the chemical potential of atom i. In thermodynamics, the chemical potential μ_i is defined as the change of Gibbs free energy with respect to a change of the particle number n of a species i at a constant temperature, pressure, and particle number of the other components,

$$\mu_i = \left(\frac{\partial G}{\partial n_i}\right)_{T,P,n_j(j\neq i)}. \quad (9)$$

For calculations in the solid phase, the temperature and pressure dependence are ignored with acceptable accuracy, and the chemical potential is set to be the calculated total energy per atom at $T = 0$ K[48,49]. Hence, for the ML WSe_2 system, the chemical potential of W and Se are represented by,

$$\frac{E(ML\, WSe_2)}{n_{WSe_2}} = \mu_W + 2\mu_{Se} = \mu_{WSe_2}, \quad (10)$$

where $E(ML\, WSe_2)$ is the calculated total energy of the ML WSe_2, and n_{WSe_2} is the number of WSe_2 units. Equation (S10) also shows that μ_W or μ_{Se} are variable with a constraint. Conventionally, the calculated total energy per atom of the elementary phase of a species i at $T =$ 0 K, i.e., $\mu_i(bulk)$, is the reference and upper bound of the chemical potential μ_i. When μ_i is equal to $\mu_i(bulk)$ in a mixture, it means that the i element is excess and its bulk starts to form[50]. Bounds on the chemical potentials of our WSe_2 system are:

$$\mu_W \le \mu_W(bulk) = \frac{E_W(bulk)}{n_w}, \tag{11}$$

$$\mu_{Se} \le \mu_{Se}(bulk) = \frac{E_{Se}(bulk)}{n_{Se}}, \tag{12}$$

where $E(W\ bulk)$ and $E(Se\ bulk)$ are the total energy of bulk W with the body-centered cubic structure and bulk Se with the trigonal structure[44], respectively. From a calculation point of view, chemical potentials vary as different atomic constituents or phases due to the different total energy. With the elementary phases as references, the amount of chemical potential change is equal to the calculated enthalpy at T = 0 K. The enthalpy calculation of the ML WSe_2 is given by,

$$\Delta H_{WSe_2} = \frac{E(ML\ WSe_2)}{n_{WSe_2}} - \frac{E_W(bulk)}{n_W} - 2 \times \frac{E_{Se}(bulk)}{n_{Se}}, \tag{13}$$

which can also be represented by,

$$\Delta H_{WSe_2} = \mu_W + 2\mu_{Se} - \mu_W(bulk) - 2\mu_{Se}(bulk) = \Delta\mu_W + 2\Delta\mu_{Se}, \tag{14}$$

where we have defined the change of the chemical potential from its bulk reference as,

$$\Delta\mu_i = \mu_i - \mu_i(bulk). \tag{15}$$

When combined with the above-mentioned equations, the lower bounds can be determined and the ranges of μ_W and μ_{Se} can be given by,

$$\Delta \mathrm{H}_{WSe_2} \le \Delta\mu_W \le 0, \tag{16}$$

and,

$$\frac{\Delta \mathrm{H}_{WSe_2}}{2} \le \Delta\mu_{Se} \le 0. \tag{17}$$

Since our system is the ML WSe_2, the relation of equation (S14) should be held in thermodynamic equilibrium. Different values of μ_W and μ_{Se} can be regarded as different conditions of W and Se ratio in the experiment. The calculated range of $\Delta\mu_{Se}$ spans from 0 to -0.63 eV for forming the ML WSe_2. When $\Delta\mu_{Se}$ is close to 0 eV, it represents a Se-rich (W-poor) condition; when $\Delta\mu_{Se}$ is close to -0.63 eV, it represents a Se-poor (W-rich) condition.

4–7. Convergence of adsorption and formation energy. For testing the convergence of the CO_2 adsorption energies, larger 4 × 9 nanoribbon models were used (Supplementary Note Fig. 6). A CO_2 molecule was introduced to interact with one of the 4 × 9 nanoribbon models at one time;

for a brief representation, the optimized results were represented together in Supplementary Note Fig. 6 a and b. Compared to the results by using 4 × 9 nanoribbon models, the CO_2 adsorption configurations are similar, the $E_b^{CO_2}$ for the CO_2 adsorption on $ZZ_{W,II}$, ZZ_{Se}, and AC edges are the same, and the $E_b^{CO_2}$ for the CO_2 adsorption on An_{Se} and An_w edges are 0.01 eV and 0.03 eV, respectively, larger when the smaller nanoribbon models were used (Supplementary Fig. 21).

Supplementary Note Table 1 shows the convergence test of defect formation energies on ZZ_{Se} and An_{Se} edges. For the ZZ_{Se} and An_{Se} edges, the differences of $\Delta\varepsilon_f$ are less than 0.04 and 0.15 eV between the 4 × 4 and the 4 × 9 models, respectively. Supplementary Note Table 2 shows a comparison of the formation energies of the in-plane defect between the nanoribbon and monolayer models. The formation energies of V_{Se} were converged. However, the formation energies of W_{Se} defects are more sensitive to the type of edges and the size of nanoribbon models. The $\Delta\varepsilon_f$ of W_{Se} defect on the basal plane of 4 × 9 nanoribbon model in Supplementary Note Fig. 6b is close to the 4 × 4 monolayer model. The $\Delta\varepsilon_f$ of W_{Se} defect on the basal plane of 4 × 9 nanoribbon model in Supplementary Note Fig. 6a is about 0.53 eV lower than that of the 4 × 4 monolayer model. We have extended the nanoribbon model in Supplementary Note Fig. 6a to a 4 × 12 nanoribbon model, and the difference is reduced to 0.29 eV. Although a larger nanoribbon model is needed to converge the formation energy of the W_{Se} defect on the basal plane, it does not affect the trend of formation energies plotted in Fig. 2d in the manuscript and Supplementary Fig. 15.

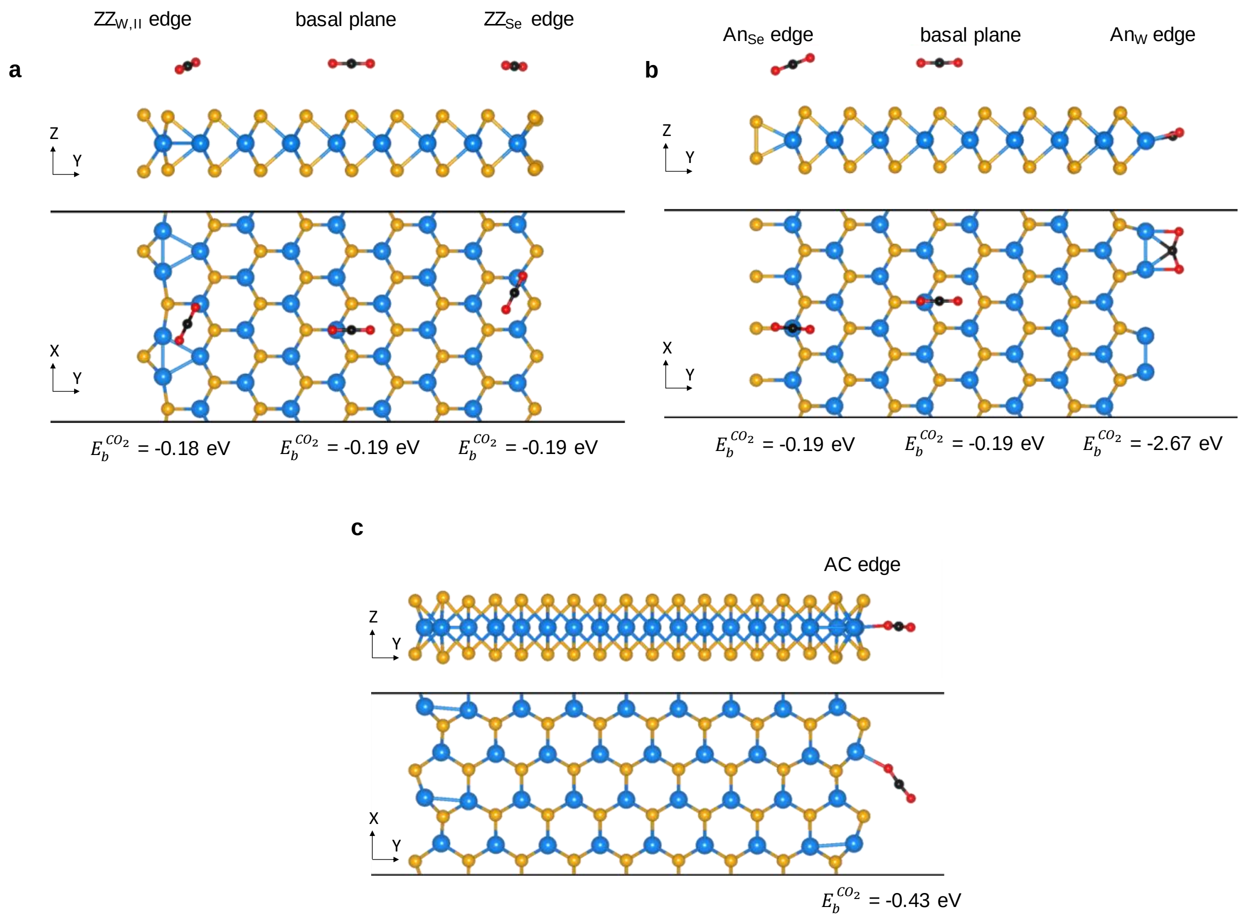

Supplementary Note Fig. 6 CO_2 adsorption on the basal plane and edges of ML WSe_2. The most stable configuration and the corresponding binding energy ($E_b^{CO_2}$) of CO_2 adsorption on 4 × 9 nanoribbon models for **a** ZZ, **b** An, and **c** AC edges, respectively. A CO_2 molecule was introduced to interact with one of the 4 × 9 nanoribbon models at one time; for a brief representation, the optimized results were represented together in (a) and (b). Blue- and dark orange- and black- and red-filled circles stand for W, Se, C, and O atoms, respectively.

Supplementary Note Table 2 The convergence test for the defect formation energies. Formation energies (calculated from $\Delta\mu_{Se}$= -0.63 to 0 eV) of the edge defects on 4 × 4 and 4 × 9 nanoribbons for ZZ_{Se} and An_{Se} edges.

Defect on ZZ_{Se}	$\Delta\varepsilon_f$ (eV) of 4 × 4 nanoribbon	$\Delta\varepsilon_f$ (eV) of 4 × 9 nanoribbon	Defect on An_{Se}	$\Delta\varepsilon_f$ (eV) of 4 × 4 nanoribbon	$\Delta\varepsilon_f$ (eV) of 4 × 9 nanoribbon
$ZZ_{Se}+V_{2Se}$	1.77–3.02	1.81–3.06	$An_{Se}+V_{2Se}$	1.34–2.59	1.40–2.66
$ZZ_{Se}+W_{Se,I}$	2.37–4.25	2.34–4.23	$An_{Se}+W_{Se,I}$	1.84–3.73	1.69–3.58
$ZZ_{Se}+W_{Se,III}$	2.95–4.84	2.93–4.82	$An_{Se}+W_{Se,III}$	4.07–5.95	4.20–6.09
$ZZ_{Se}+W_{add}$	2.13–3.38	2.10–3.36	$An_{Se}+W_{add}$	1.01–2.26	1.00–2.26

Supplementary Note Table 3 A comparison of the in-plane defect formation energies between the nanoribbon and monolayer models. Formation energies (calculated from $\Delta\mu_{Se}$= -0.63 to 0 eV) of the V_{Se} and W_{Se} in-plane defects on the 4 × 4 monolayer model and 4 × 9 nanoribbon models for ZZ and An edges.

In-plane defects	$\Delta\varepsilon_f$ (eV) of 4 × 9 nanoribbon model in Supplementary Note Fig. 6a	$\Delta\varepsilon_f$ (eV) of 4 × 9 nanoribbon model in Supplementary Note Fig. 6b	$\Delta\varepsilon_f$ (eV) of 4 × 4 monolayer model from Supplementary Fig. 15
V_{Se}	2.01–2.64	2.01–2.64	2.05–2.68
W_{Se}	5.12–7.00	5.57–7.45	5.65–7.53

Supplementary Note 5. Ag photodeposition and nanoscale redox mapping

5–1. Photodeposition of Ag nanoparticles. We performed a photodeposition of Ag nanoparticle experiment, to study the most probable charge transfer sites to the environment. A dilute 1 nM $AgNO_3$ aqueous solution and CH_3OH (20% v/v as the hole scavenger) was used as the electrolyte under a solar simulator for 1 h. Transferred electron to Ag ion results in the deposition of Ag nanoparticles on the surface.

5–2. Atomic force microscopy-scanning electrochemical microscopy. A Bruker Dimension Icon, in a PeakForce scanning electrochemical microscopy (SECM) module equipped with CHI760D electrochemical analyzer, was used to provide nanoscale redox mapping at the ML WSe_2–liquid interface. For the atomic force microscopy (AFM)-SECM study, we used ML WSe_2 grown by atmospheric-pressure chemical vapor deposition (See Supplementary Note 1). In this cell, a commercialized nanoelectrode probe (Bruker), 0.3 mm Pt, and 0.2 mm Ag wires were used

as the working, counter, and pseudo-reference electrodes, respectively. The nanoelectrode probe was coated with dielectric materials except for the Pt tip apex of approximately 200 nm in height and 25 nm in end-probe radius[51,52]. The flake was immersed in an aqueous solution containing 10 mM hexaammineruthenium (iii) chloride ($[Ru(NH_3)_6]Cl_3$) and 0.1 M potassium nitrate (KNO_3) as a reversible redox mediator a supporting electrolyte, respectively. Before the SECM measurement, the nanoelectrode probe was tested by performing six cyclic voltammograms (0.02 V s^{-1}, from 0 to -0.5 V versus pseudo-reference electrode). The potential of the working electrode was fixed at -0.4 V for the reduction of Ru^{3+} to Ru^{2+}, while the sample was left unbiased (0 V) for the oxidation of Ru^{2+} back to Ru^{3+}. Finally, PeakForce SECM imaging scans the probe under the main (with the tip 25 nm to the flake) and lift scans (with the tip 100 nm above the flake) surface. It should be noted that the lateral resolution of our SECM measurement is about ± 0.2 μm. Supplementary Note Fig. 7 shows that the main feedback current starts to increase when the tip reaches a distance of about 0.2 μm from the Pt layer due to the diffusion of the reactant in the electrolyte. Lift feedback current shows an order lower signal due to the larger distance between the tip and the substrate.

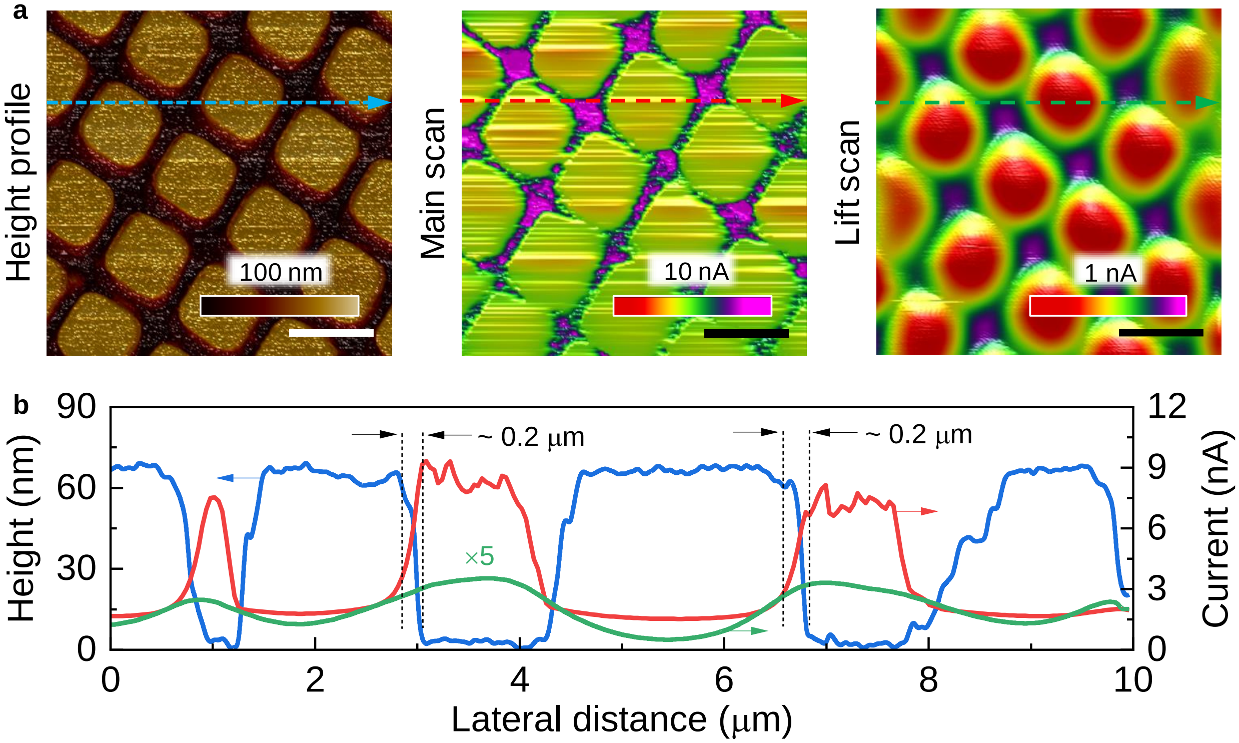

Supplementary Note Fig. 7 AFM-SECM feedback mapping of Pt strands separated by square Si_3N_4 islands. a AFM height profile measured in the liquid environment, background normalized SECM (main and lift scans) feedback maps. Scale bar, 2 μm. **b** Line-scan analysis of topography (blue line), tip current from main scan (red line), and tip current from lift scan (green line).

Further, we have used analytical approximations for fitting the positive and negative normalized feedback currents ($\frac{I}{I_0}$, where I_0 is the steady-state current when the tip is far from the surface)[53,54]:

$$\frac{I^+}{I_0} = K_1^+ + \frac{K_2^+}{\left(\frac{z}{a}\right)} + K_3^+ e^{\frac{K_4^+}{\left(\frac{z}{a}\right)}}, \tag{18}$$

$$\frac{I^-}{I_0} = \left(K_1^- + \frac{K_2^-}{\left(\frac{z}{a}\right)} + K_3^- e^{\frac{K_4^-}{\left(\frac{z}{a}\right)}}\right)^{-1}, \tag{19}$$

where $K_1^\pm$, $K_2^\pm$, $K_3^\pm$, and $K_4^\pm$ are dimensionless constants. And $\frac{z}{a}$ (where a is the radius of the Pt tip) is the normalized tip–surface distance.

Supplementary Note 6. Photocatalytic CO_2 reduction

6–1. Photocatalytic setup. Photocatalytic (PC) CO_2 reduction experiments were performed using a home-built stainless steel reactor at room temperature. Before starting the PC experiment, the reactor was degassed at 80 °C and 150 °C for 24 and 3 h, respectively, followed by blowing with an N_2 gun for several minutes. Then, ultrapure CO_2 gas was purged into the reactor at a flow rate of 90 and 30 sccm for 10 and 30 min, respectively, to provide a constant humidity level and equilibrium gas adsorption–desorption inside the reactor. After that, a commercial 150 W Xe lamp (> 320 nm, AM 1.5G, 100 mW cm^{-2}) was placed as a light source directly above the quartz window of the reactor. The background signal of the CH_4 molecules was measured by injecting Ar gas several times to reach a constant peak area, and finally, subtracted from the total yield. The PC reaction was terminated after each reaction cycle to avoid the adsorbed product on the catalyst surface. Then, the photocatalyst was degassed by N_2 flush followed by keeping in a vacuum box for 24 h. We have further performed a $^{13}CO_2$ isotope test by using RT-Msieve 5A (15m I.D.: 0.25 mm) column at 35 °C under a He flows at a rate of 1 mL min^{-1}. Supplementary Note Fig. 8 shows the presence of both $^{13}CH_4$ and also $^{12}CH_4$. The presence of $^{12}CH_4$ can be due to the dissolved $^{12}CO_2$ in water or carbonate species which pre-adsorbed on the surface under the ambient atmosphere[55,56].

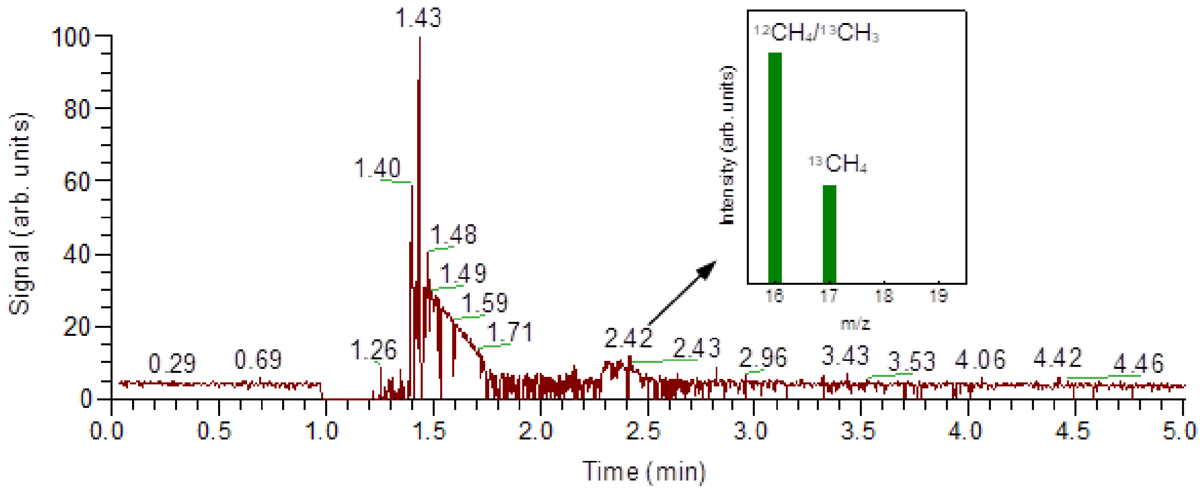

Supplementary Note Fig. 8 Gas chromatography–mass spectrometry of the CH_4 product. The gas chromatograph (GC) shows the presence of methane as the product. Inset shows the GC-Mass, illustrating the presence of $^{13}CH_4$ with m/z = 17. The fragment at m/z = 16 can be assigned to $^{13}CH_3$ and $^{12}CH_4$.

Therefore, we have also performed the blank tests[57]: (i) With WSe_2 photocatalyst/with CO_2/without light (with the production of Y_{b1}), (ii) With WSe_2 photocatalyst/without CO_2/with light (with the production of Y_{b2}), and (iii) Without WSe_2 photocatalyst/with CO_2/with light (with the production of Y_{b3}). Finally, the values of products measured in the blank experiments have subtracted from the product yield in the photocatalytic reaction as,

$$Y = Y_{exp} - Y_{b1} - Y_{b2} - Y_{b3}. \quad (20)$$

Additionally, helium ionization detector (HDI) and GC-MS were used to check the presence of CO and qualitatively measure the oxidation products.

6–2. Calculation of internal quantum efficiency. Internal quantum efficiency (IQE) is defined by the ratio of the number of electrons used for CH_4 production to the total absorbed photon flux ($\bar{\phi}_A$, see Supplementary Note 3),

$$IQE = \frac{8 \times Y \times N_A}{\bar{\phi}_A \times t}, \quad (21)$$

where Y, N_A, and t are the total CH_4 yield, Avogadro's number, and irradiation time, respectively. IQE is independent of the total area (S). Further, the total product (Y) was modeled with $Y = (f_b + \frac{f_e}{\bar{P}})S_t$ where f_b and f_e are fitting factors depicting the contributions of the basal plane and edge, respectively, and S_t is the total area. Notably, these fitting factors are obtained by fitting the IQE, which is independent of S_t,

$$IQE = \gamma(f_b + \frac{f_e}{\bar{P}}), \quad (22)$$

where γ is a constant coefficient that is a function of the number of electrons used for CH_4 production, the total adsorbed photon flux, and Avogadro's number. So, the edge contribution in the final product is proportional to the perimeter-dependent factor $\frac{\frac{f_e}{\bar{P}}}{f_b + \frac{f_e}{\bar{P}}}$.

6–3. Calculation of consumed electron rates. The turnover frequency (TOF) is defined as the ratio of total number of CH_4 molecules per second per the number of active sites at the edge ($S \times N_{act.\ sites}^{edge}$), as expressed by the following equation:

$$TOF_{CH_4}^{edge} = \frac{(\frac{Y}{t})N_A}{S \times N_{act.\ sites}^{edge}}, \quad (23)$$

where S is the total area of the flakes. Edge atoms have a size-depended density of $N_{act.\ sites}^{edge} \lesssim \frac{2\times10^{13}}{P}$. By adding the edge contribution, i.e. $\frac{\frac{f_e}{P}}{f_b+\frac{f_e}{P}}$, to the equation (S11), $TOF_{CH_4}^{edge}$ was calculated to be about 0.48 ± 0.04 s^{-1} from the following equation:

$$TOF_{CH_4}^{edge} \approx \frac{3.0\times10^4 f_e}{t}, \quad (24)$$

where f_e = 0.23. Similarly, the $TOF_{CH_4}^{basal}$ of the intrinsic defects on the basal plane was calculated to be about 0.0034 ± 0.0009 s^{-1} from the following equation:

$$TOF_{CH_4}^{basal} \approx \frac{1.2\times10^4 f_b}{t}, \quad (25)$$

where f_b = 0.007 f_e. To compare the reported catalysts with different products, we defined the consumed electron rate (R_e), total number of consumed electrons per second per the number of active sites:

$$R_e = N_e \times TOF_{product}, \quad (26)$$

where N_e is the number of reacted electrons in a reduction reaction. For example, N_e are 2 and 8 for H^+ to H_2 and CO_2 to CH_4 reactions, respectively.

Supplementary Note 7. Illustration of the relaxed configurations

VESTA (3.5.7) software[58] was used to draw the relaxed configurations.

Supplementary References

1 Temple, P. A. & Hathaway, C. E. Multiphonon Raman-Spectrum of Silicon. *Phys. Rev. B: Condens. Matter* **7**, 3685-3697 (1973).

2 Uchinokura, K., Sekine, T. & Matsuura, E. Critical-point analysis of the two-phonon Raman spectrum of silicon. *J. Phys. Chem. Solids* **35**, 171-180 (1974).

3 Fan, X. *et al.* Nonlinear photoluminescence in monolayer WS_2: parabolic emission and excitation fluence-dependent recombination dynamics. *Nanoscale* **9**, 7235-7241 (2017).

4 Mak, K. F. *et al.* Tightly bound trions in monolayer MoS_2. *Nat. Mater.* **12**, 207-211 (2013).

5 Steinhoff, A. *et al.* Efficient excitonic photoluminescence in direct and indirect band gap monolayer MoS_2. *Nano Lett.* **15**, 6841-6847 (2015).

6 Vina, L., Logothetidis, S. & Cardona, M. Temperature-dependence of the dielectric function of germanium. *Phys. Rev. B: Condens. Matter* **30**, 1979-1991 (1984).

7 Huang, J., Hoang, T. B. & Mikkelsen, M. H. Probing the origin of excitonic states in monolayer WSe_2. *Sci. Rep.* **6**, 22414 (2016).

8 Gao, Y. *et al.* Ultrafast growth of high-quality monolayer WSe_2 on Au. *Adv. Mater.* **29**, 1700990 (2017).

9 Yang, D., Fan, X., Zhang, F., Hu, Y. & Luo, Z. Electronic and magnetic properties of defected monolayer WSe_2 with vacancies. *Nanoscale Res. Lett.* **14**, 192 (2019).

10 Kamal Hussien, M. *et al.* Metal-free four-in-one modification of g-C_3N_4 for superior photocatalytic CO_2 reduction and H_2 evolution. *Chem. Eng. J.* **430**, 132853 (2022).

11 Ali, S. *et al.* Sustained, photocatalytic CO_2 reduction to CH_4 in a continuous flow reactor by earth-abundant materials: Reduced titania-Cu_2O Z-scheme heterostructures. *Appl. Catal., B* **279**, 119344 (2020).

12 Dao, X. Y. *et al.* Structure-dependent iron-based metal-organic frameworks for selective CO_2-to-CH_4 photocatalytic reduction. *J. Mater. Chem. A* **8**, 25850-25856 (2020).

13 Billo, T. *et al.* A mechanistic study of molecular CO_2 interaction and adsorption on carbon implanted SnS2 thin film for photocatalytic CO_2 reduction activity. *Nano Energy* **72**, 104717 (2020).

14 Wang, C. J. *et al.* All-solid-state Z-scheme photocatalysts of g-C_3N_4/Pt/macroporous-(TiO_2@carbon) for selective boosting visible-light-driven conversion of CO_2 to CH_4. *J. Catal.* **389**, 440-449 (2020).

15 Sorcar, S. *et al.* CO_2, water, and sunlight to hydrocarbon fuels: a sustained sunlight to fuel (Joule-to-Joule) photoconversion efficiency of 1%. *Energy Environ. Sci.* **12**, 2685-2696 (2019).

16 Li, Y. *et al.* TiO_{2-x}/CoO_x photocatalyst sparkles in photothermocatalytic reduction of CO_2 with H_2O steam. *Appl. Catal., B* **243**, 760-770 (2019).

17 Shankar, R. *et al.* Porous boron nitride for combined CO_2 capture and photoreduction. *J. Mater. Chem. A* **7**, 23931-23940 (2019).

18 Wang, S. *et al.* Porous hypercrosslinked polymer-TiO_2-graphene composite photocatalysts for visible-light-driven CO_2 conversion. *Nat. Commun.* **10**, 676 (2019).

19 Li, X. D. *et al.* Selective visible-light-driven photocatalytic CO_2 reduction to CH_4 mediated by atomically thin $CuIn_5S_8$ layers. *Nat. Energy* **4**, 690-699 (2019).

20 Sorcar, S. *et al.* High-rate solar-light photoconversion of CO_2 to fuel: controllable transformation from C_1 to C_2 products. *Energy Environ. Sci.* **11**, 3183-3193 (2018).

21 Jiang, Z. F. *et al.* Nature-based catalyst for visible-light-driven photocatalytic CO_2 reduction. *Energy Environ. Sci.* **11**, 2382-2389 (2018).

22 Sorcar, S., Hwang, Y. J., Grimes, C. A. & In, S. I. Highly enhanced and stable activity of defect-induced titania nanoparticles for solar light-driven CO_2 reduction into CH_4. *Mater. Today* **20**, 507-515 (2017).

23 Jiao, X. *et al.* Partially oxidized SnS_2 atomic layers achieving efficient visible-light-driven CO_2 reduction. *J. Am. Chem. Soc.* **139**, 18044-18051 (2017).

24 Fang, B. Z., Xing, Y. L., Bonakdarpour, A., Zhang, S. C. & Wilkinson, D. P. Hierarchical CuO-TiO_2 hollow microspheres for highly efficient photodriven reduction of CO_2 to CH_4. *ACS Sustainable Chem. Eng.* **3**, 2381-2388 (2015).

25 Jin, J., Yu, J., Guo, D., Cui, C. & Ho, W. A hierarchical Z-scheme CdS-WO_3 photocatalyst with enhanced CO_2 reduction activity. *Small* **11**, 5262-5271 (2015).

26 Hu, J. T. *et al.* Sulfur vacancy-rich MoS_2 as a catalyst for the hydrogenation of CO_2 to methanol. *Nat. Catal.* **4**, 242-250 (2021).

27 Padmajan Sasikala, S. *et al.* Longitudinal unzipping of 2D transition metal dichalcogenides. *Nat. Commun* **11**, 5032 (2020).

28 Li, H. *et al.* Activating and optimizing MoS_2 basal planes for hydrogen evolution through the formation of strained sulphur vacancies. *Nat. Mater.* **15**, 48-53 (2016).

29 Asadi, M. *et al.* Nanostructured transition metal dichalcogenide electrocatalysts for CO_2 reduction in ionic liquid. *Science* **353**, 467-470 (2016).

30 Zhang, J., Liu, S., Liang, H., Dong, R. & Feng, X. Hierarchical transition-metal dichalcogenide nanosheets for enhanced electrocatalytic hydrogen evolution. *Adv. Mater.* **27**, 7426-7431 (2015).

31 Kong, D. *et al.* Synthesis of MoS_2 and $MoSe_2$ films with vertically aligned layers. *Nano Lett.* **13**, 1341-1347 (2013).

32 Jaramillo, T. F. *et al.* Hydrogen evolution on supported incomplete cubane-type $[Mo_3S_4]^{4+}$ electrocatalysts. *J. Phys. Chem. C* **112**, 17492-17498 (2008).

33 Jaramillo, T. F. *et al.* Identification of active edge sites for electrochemical H_2 evolution from MoS_2 nanocatalysts. *Science* **317**, 100-102 (2007).

34 Zhou, H. *et al.* Large area growth and electrical properties of p-type WSe_2 atomic layers. *Nano Lett.* **15**, 709-713 (2015).

35 Leonard, J. *et al.* High-throughput time-correlated single photon counting. *Lab Chip* **14**, 4338-4343 (2014).

36 Wang, Y. J. *et al.* Wafer-scale synthesis of monolayer WSe_2: A multi-functional photocatalyst for efficient overall pure water splitting. *Nano Energy* **51**, 54-60 (2018).

37 Li, Y. *et al.* Measurement of the optical dielectric function of monolayer transition-metal dichalcogenides: MoS_2, $MoSe_2$, WS_2, and WSe_2. *Phys. Rev. B: Condens. Matter* **90**, 205422 (2014).

38 Kresse, G. & Furthmuller, J. Efficient iterative schemes for ab initio total-energy calculations using a plane-wave basis set. *Phys. Rev. B Condens. Matter.* **54**, 11169-11186 (1996).

39 Kresse, G. & Joubert, D. From ultrasoft pseudopotentials to the projector augmented-wave method. *Phys. Rev. B Condens. Matter.* **59**, 1758-1775 (1999).

40 Perdew, J. P., Burke, K. & Ernzerhof, M. Generalized gradient approximation made simple. *Phys. Rev. Lett.* **77**, 3865-3868 (1996).

41 Grimme, S., Antony, J., Ehrlich, S. & Krieg, H. A consistent and accurate ab initio parametrization of density functional dispersion correction (DFT-D) for the 94 elements H-Pu. *J. Chem. Phys.* **132**, 154104 (2010).

42 Schutte, W. J., Deboer, J. L. & Jellinek, F. Crystal-structures of tungsten disulfide and diselenide. *J. Solid State Chem.* **70**, 207-209 (1987).

43 Heyd, J., Scuseria, G. E. & Ernzerhof, M. Hybrid functionals based on a screened Coulomb potential. *J. Chem. Phys.* **118**, 8207-8215 (2003).

44 Hu, G., Fung, V., Sang, X., Unocic, R. R. & Ganesh, P. Predicting synthesizable multi-functional edge reconstructions in two-dimensional transition metal dichalcogenides. *npj Comput. Mater.* **6**, 44 (2020).

45 Cui, P. *et al.* Contrasting structural reconstructions, electronic properties, and magnetic orderings along different edges of zigzag transition metal dichalcogenide nanoribbons. *Nano Lett.* **17**, 1097-1101 (2017).

46 Tang, W., Sanville, E. & Henkelman, G. A grid-based Bader analysis algorithm without lattice bias. *J. Phys. Condens. Matter.* **21**, 084204 (2009).

47 Zhou, W. *et al.* Intrinsic structural defects in monolayer molybdenum disulfide. *Nano Lett.* **13**, 2615-2622 (2013).

48 Qian, G. X., Martin, R. M. & Chadi, D. J. First-principles study of the atomic reconstructions and energies of Ga- and As-stabilized GaAs(100) surfaces. *Phys. Rev. B Condens. Matter.* **38**, 7649-7663 (1988).

49 Zhang, S. B. & Northrup, J. E. Chemical potential dependence of defect formation energies in GaAs: Application to Ga self-diffusion. *Phys. Rev. Lett.* **67**, 2339-2342 (1991).

50 Freysoldt, C. *et al.* First-principles calculations for point defects in solids. *Rev. Mod. Phys.* **86**, 253-305 (2014).

51 Nellist, M. R. *et al.* Atomic force microscopy with nanoelectrode tips for high resolution electrochemical, nanoadhesion and nanoelectrical imaging. *Nanotechnology* **28**, 095711 (2017).

52 Du, H. Y. *et al.* Nanoscale redox mapping at the MoS_2-liquid interface. *Nat. Commun.* **12**, 1321 (2021).

53 Kwak, J. & Bard, A. J. Scanning electrochemical microscopy - Theory of the feedback mode. *Anal. Chem.* **61**, 1221-1227 (1989).

54 Amphlett, J. L. & Denuault, G. Scanning electrochemical microscopy (SECM): An investigation of the effects of tip geometry on amperometric tip response. *J. Phys. Chem. B* **102**, 9946-9951 (1998).

55 Park, H., Ou, H.-H., Colussi, A. J. & Hoffmann, M. R. Artificial photosynthesis of C_1–C_3 hydrocarbons from Water and CO_2 on titanate nanotubes decorated with nanoparticle elemental Copper and CdS quantum dots. *J. Phys. Chem. A* **119**, 4658-4666 (2015).

56 Kharade, A. K. & Chang, S.-m. Contributions of abundant hydroxyl groups to extraordinarily high photocatalytic activity of amorphous titania for CO_2 reduction. *J. Phys. Chem. C* . **124**, 10981-10992 (2020).

57 Moustakas, N. G. & Strunk, J. Photocatalytic CO_2 reduction on TiO_2-based materials under controlled reaction conditions: systematic insights from a literature study. *Chem. Eur. J.* **24**, 12739-12746 (2018).

58 Momma, K. & Izumi, F. VESTA 3 for three-dimensional visualization of crystal, volumetric and morphology data. *J. Appl. Crystallogr.* **44**, 1272-1276 (2011).
